# TBK1 Induces the Formation of Optineurin Filaments That Condensate with Polyubiquitin and LC3 for Cargo Sequestration

**DOI:** 10.1002/advs.202509927

**Published:** 2025-12-17

**Authors:** Maria G. Herrera, Lena Kühn, Lisa Jungbluth, Verian Bader, Laura J. Krause, David Kartte, Elias Adriaenssens, Sascha Martens, Jörg Tatzelt, Carsten Sachse, Konstanze F. Winklhofer

**Affiliations:** ^1^ Department Molecular Cell Biology Institute of Biochemistry and Pathobiochemistry Ruhr University Bochum 44801 Bochum Germany; ^2^ Faculty of Exact and Natural Sciences Institute of Biosciences Biotechnology and Translational Biology (iB3) University of Buenos Aires Intendente Güiraldes 2160, Ciudad Universitaria Buenos Aires C1428EGA Argentina; ^3^ Ernst‐Ruska Centre for Microscopy and Spectroscopy with Electrons ER‐C‐3/Structural Biology, Forschungszentrum Jülich 52425 Jülich Germany; ^4^ Department of Biology Heinrich Heine University Universitätsstr. 1 40225 Düsseldorf Germany; ^5^ Max Perutz Labs, Vienna Biocenter Campus (VBC) Vienna 1030 Austria; ^6^ Max Perutz Labs, Department of Biochemistry and Cell Biology University of Vienna Vienna 1030 Austria; ^7^ Department Biochemistry of Neurodegenerative Diseases Institute of Biochemistry and Pathobiochemistry Ruhr University Bochum 44801 Bochum Germany; ^8^ Cluster of Excellence RESOLV Ruhr University Bochum 44801 Bochum Germany

**Keywords:** autophagy, Optineurin, phase separation, TBK1, ubiquitin

## Abstract

Optineurin is an autophagy receptor that plays an important role in the selective degradation of mitochondria, protein aggregates, and intracellular pathogens. It recognizes ubiquitylated cargo by its ubiquitin‐binding in ABIN and NEMO (UBAN) domain and recruits the autophagic machinery through its LC3‐interacting region (LIR) domain. Phosphorylation of Optineurin by TANK‐binding kinase 1 (TBK1) increases the binding of Optineurin to both ubiquitin chains and lipidated microtubule‐associated protein light chain 3 (LC3). Optineurin has been reported to form foci at ubiquitylated cargo, but the underlying mechanism and how these foci are linked to selective autophagy has remained largely unknown. This study shows that phosphorylation of Optineurin by TBK1 induces the formation of filaments that phase separate upon binding to linear polyubiquitin. LC3 anchored to unilamellar vesicles co‐partitions into Optineurin/polyubiquitin condensates, resulting in the local deformation of the vesicle membrane. Thus, the condensation of filamentous Optineurin with ubiquitylated cargo promotes the nucleation of cargo and its subsequent alignment with LC3‐positive nascent autophagosomes, suggesting that co‐condensation processes ensure directionality in selective autophagy.

## Graphical abstract

Phosphorylation of Optineurin by TBK1 induces the formation of filaments that condensate upon binding to linear polyubiquitin. Membrane‐anchored LC3 partitions into these condensates, suggesting that phase separation of filamentous Optineurin with ubiquitylated cargo promotes the sequestration of cargo and its subsequent alignment with LC3‐positive nascent autophagosomes.

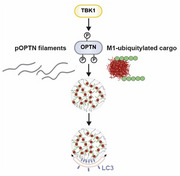

## 1 Introduction

Selective autophagy allows the degradation of diverse cellular components, including damaged and dysfunctional organelles, protein aggregates and intracellular pathogens, to maintain cellular homeostasis.^[^ ^1^, ^2^, ^3^, ^4^, ^5^, ^6^, ^7^, ^8^, ^9^ ^]^ During this process, the cargo is engulfed within a double‐membrane structure generated *de novo*, known as the autophagosome. Biogenesis of autophagosomes occurs at the cargo by forming the phagophore, which is labelled by lipidated microtubule‐associated protein light chain 3 (LC3)/gamma‐ aminobutyric acid receptor‐associated protein (GABARAP) proteins. After segregation of the cargo, the double membrane closes and the autophagosome fuses with the endosomal‐lysosomal system to degrade cargo along with the inner membrane. Selectivity in this process is achieved by labelling the cargo for autophagosomal degradation and binding to specific cargo receptor proteins, including sequestosome 1 (p62/SQSTM1), neighbor of BRCA1 (NBR1), Tax1 binding protein 1 (TAX BP1), nuclear dot protein 52 (NDP52), and Optineurin.^[^ ^10^ ^]^ Marking of cargo for selective autophagy is usually mediated by ubiquitylation, although ubiquitin‐independent mechanisms also exist.^[^ ^11^ ^]^ Cargo receptors contain a ubiquitin‐binding domain for cargo recognition and a LC3 interacting region (LIR) domain for binding to LC3/GABARAP proteins at the phagophore. Thereby, cargo receptors link the cargo to the expanding membrane of the phagophore. Moreover, some cargo receptors have a function upstream of autophagosome biogenesis by sequestering ubiquitylated cargo into condensates. In particular, p62 and NBR1 form phase‐separated condensates upon binding to ubiquitylated cargo, which is a prerequisite for the efficient recruitment of the autophagic machinery.^[^ ^12^ ^−^ ^16^ ^]^

**Figure 1:**
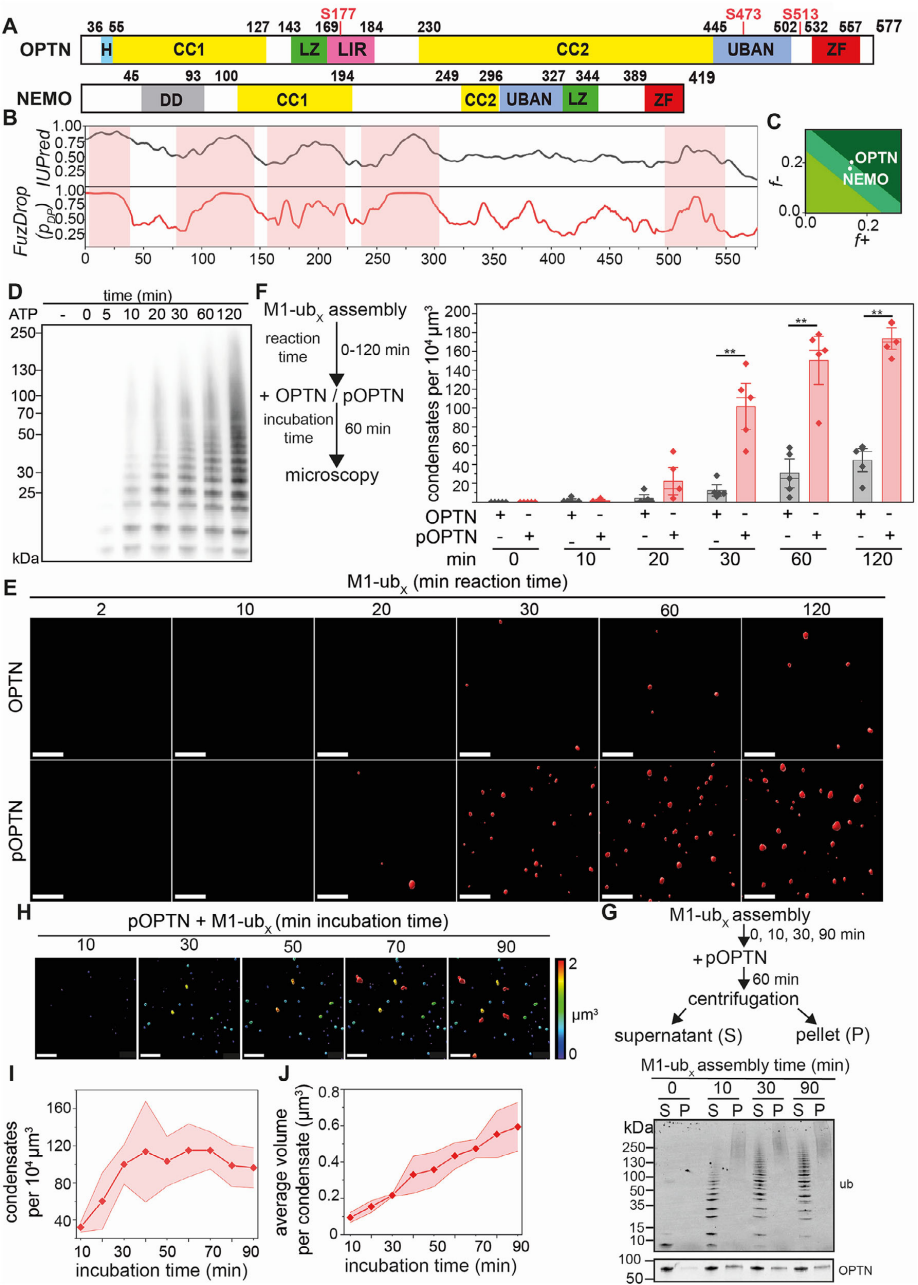
Phosphorylation by TBK1 regulates phase separation of Optineurin induced by M1‐linked polyubiquitin. A) Domain structure of Optineurin (OPTN) and NEMO. H: helix‐loop‐helix, CC1: coiled‐coil domain 1, CC2: coiled‐coil domain 2, LZ: leucine zipper, LIR: LC3‐interacting region, UBAN: ubiquitin‐binding domain in ABIN proteins and NEMO, ZF: zinc finger, DD: dimerization domain. Serine residues phosphorylated by TBK1 are shown in red. B) Identification of IDRs in Optineurin using IUPred and FuzDrop. Residues with high disorder propensity are indicated by red shadows. C) Analysis of the phase separation propensity of OPTN and NEMO using CIDER. The *x*‐axis represents the fraction of negatively charged residues, whereas the *y*‐axis corresponds to the fraction of positively charged residues. D) In vitro assembly of M1‐polyubiqutin chains (M1‐ub_x_) over different time periods (reaction time). The reaction was initiated by adding ATP and stopped by acidification. M1‐ub_x_ chains were analysed by immunoblotting using the specific M1‐polyubiquitin antibody 1E3. E,F) Formation of Optineurin condensates is increased with the reaction time of M1‐ub_x_ assembly. E) Fluorescence microscopy of condensates formed by Optineurin (OPTN) (5 µM) or Optineurin phosphorylated by TBK1 (pOPTN) (5 µM) in the presence of M1‐ub_x_ (40 µM) generated over different time periods. Images are shown by 3D projections of the fluorescence images. Scale bar: 5 µm. F. Quantification of the number in condensates formed in the presence of M1‐ub_x_ and OPTN and p‐OPTN generated over different time periods (reaction time) corresponding to E. Data represent the mean ± standard deviation (SD) of five independent experiments. The statistical analysis was performed using a Mann‐Whitney test (**p = 0.0079, n = 5). G) Sedimentation assay of pOPTN condensates formed in the presence of M1‐ub_x_ assembled over time (0, 10, 30, and 90 min reaction time). pOPTN (5 µM) was incubated with M1‐ub_x_ (40 µM) for 60 min. After centrifugation, the supernatant (S) and pellet (P) fractions were analyzed by immunoblotting. The ubiquitin chains were analyzed using an anti‐ubiquitin antibody (P4D1). H) The number and average volume of pOPTN condensates formed in the presence of M1‐ub_x_ increase over time. 3D reconstruction of fluorescence microscopy of pOPTN condensates formed in the presence of M1‐ub_x_ (reaction time 120 min) over time (incubation time). The size of the condensates is color‐coded. Scale bar: 7 µm. I,J) Quantification of the number (I) and average volume (J) of the pOPTN condensates formed in the presence of M1‐ub_x_ over time. The analysis is based on three independent experiments.

Human Optineurin is a ubiquitously expressed 577 amino acid protein that in addition to the selective autophagy of cellular organelles, protein aggregates and bacteria is involved in immune signaling, cell death regulation and intracellular vesicular trafficking.^[^ ^17^, ^18^, ^19^, ^20^, ^21^, ^22^, ^23^, ^24^, ^25^, ^26^, ^27^, ^28^, ^29^ ^]^ It is composed of two coiled coil motifs (CC) separated by a leucine zipper motif (LZ) and a LIR domain, a ubiquitin‐binding in ABIN and NEMO (UBAN) domain, and a C‐terminal zinc finger motif (ZF) (**Figure** **1**A). Optineurin binds preferentially to M1‐ and K63‐linked polyubiquitin.^[^ ^30^, ^31^ ^]^ Phosphorylation of serine 473 within the UBAN domain by TANK‐binding kinase 1 (TBK1) increases the binding affinity of Optineurin to ubiquitin chains and is important for the function of Optineurin in selective autophagy.^[^ ^30^, ^31^, ^32^, ^33^ ^]^ Optineurin can even induce an unconventional mechanism of autophagy initiation by using TBK1 as a UNC‐51‐like kinase 1 and 2 (ULK 1/2).^[^ ^34^ ^]^ Dysfunction of the TBK1/Optineurin pathway caused by mutations in the genes encoding TBK1 or Optineurin is linked to amyotrophic lateral sclerosis (ALS) and frontotemporal dementia (FTD).^[^ ^35^, ^36^, ^37^, ^38^ ^]^

Optineurin has been reported to form foci at cargo destined for selective autophagy.^[^ ^39^, ^40^ ^]^ However, the biochemical and biophysical mechanisms underlying this process are not well understood. Here we show that upon phosphorylation by TBK1, Optineurin forms ribbon‐like filaments. Binding of phosphorylated polymeric Optineurin to linear ubiquitin chains induces the formation of phase‐separated condensates into which membrane‐anchored LC3 co‐partitions. These data suggest that cargo sequestration and subsequent alignment with the expanding phagophore is coordinated by co‐condensation with filamentous Optineurin.

## 2 Results

### 2.1 Phosphorylation by TBK1 and Binding to M1‐Linked Ubiquitin Chains Promote Phase Separation of Optineurin

Optineurin and NEMO (NF kappa B essential modifier) share similarities in their domain structure (Figure 1A). Both proteins have a UBAN domain, which binds to linear or M1‐linked ubiquitin chains with high specificity.^[^ ^31^, ^32^, ^41^, ^42^ ^]^ We and others have observed that NEMO undergoes phase separation when it binds to M1‐linked polyubiquitin. This process is necessary for NEMO to function in NF‐κB signaling and selective autophagy.^[^ ^43^, ^44^, ^45^ ^]^ We therefore wondered if the physiological function of Optineurin is also regulated by phase separation induced by M1‐linked polyubiquitin binding. The ability to undergo phase separation is often associated with intrinsically disordered regions (IDRs).^[^ ^46^, ^47^, ^48^, ^49^ ^]^ To determine whether Optineurin contains disordered regions, we used IUPred2A, which predicts disordered residues based on the IUPred2 algorithm.^[^ ^50^ ^]^ This analysis revealed that the first 50 residues of the N‐terminal region of Optineurin, the last part of the CC1 domain, the first part of the LZ, the LIR, and the region between the UBAN domain and the ZF domain are intrinsically disordered. FuzDrop predicts the probability of spontaneous liquid‐liquid phase separation (LLPS) by calculating a global score (LLPS > 0.6), which was 0.9345 for Optineurin (Figure 1B). It also provides a sequence‐based score to identify regions that promote this process.^[^ ^51^, ^52^ ^]^ In addition, we used the Classification of Intrinsically Disordered Ensemble Regions (CIDER) for the analysis of charge distribution within IDRs.^[^ ^53^ ^]^ According to CIDER, Optineurin was located in the region of Janus sequences, which refers to collapsed or expanded proteins, whose behavior depends on factors such as salt concentration, ligand binding and cis‐interactions, and showed a low κ (κ = 0.114), indicating a uniform distribution of charges throughout the protein (Figure 1C). A similar classification was found for NEMO (Figure 1C).

To test the ability of Optineurin to undergo phase separation, we expressed human glutathione S‐transferase (GST)‐tagged Optineurin in *E. coli*. After purification, GST was cleaved by the Tobacco Etch Virus protease (TEV) and removed by affinity purification. Optineurin was then labeled by Cyanine5 (Cy5) maleimide for fluorescence‐based detection. M1‐linked polyubiquitin chains of different lengths (M1‐ub_x_) were assembled in vitro to test their role in promoting phase separation of Optineurin. To this end, we used catalytically active C‐terminal HOIP (amino acids 606 to 1072), which is the catalytic E3 ubiquitin ligase of the linear ubiquitin chain assembly complex (LUBAC), as HOIP can assemble un‐anchored “free” linear ubiquitin chains.^[^ ^54^ ^]^ C‐terminal HOIP was mixed with the E2 ubiquitin‐conjugating enzyme UBE2L3, the mouse E1 ubiquitin‐activating enzyme, and recombinant mono‐ubiquitin. The enzymatic reaction was stopped at different time points and M1‐linked ubiquitin (M1‐ub_x_) chains were analysed by immunoblotting for their quantity and length spectrum over time (Figure 1D). Fluorescence microscopy showed that Optineurin alone did not phase separate (Figure 1E,F). When we mixed Optineurin with M1‐ub_x_ chains and performed fluorescence microscopy after 60 min, a small number of Optineurin condensates was observed with M1‐ub_x_ chains generated for 30, 60, and 120 min in vitro (Figure 1E,F). In the following, we use the term “condensates” for assemblies formed by phase‐separation in vitro irrespective of their material properties, according to the current practices in the field.^[^ ^55^ ^]^ The term “foci” is used to describe protein assemblies in cells. Since phosphorylation of Optineurin by TBK1 increases the binding affinity of Optineurin to ubiquitin chains,^[^ ^30^, ^31^, ^32^, ^33^ ^]^ we phosphorylated Optineurin by purified TBK1 in vitro (Figure S1A,B, Supporting Information). After removing TBK1 by affinity chromatography, phosphorylated Optineurin (p‐Optineurin) was labelled with Cy5 (Figure S1C, Supporting Information). Then, we compared the phase separation behavior of non‐phosphorylated and phosphorylated Optineurin. P‐Optineurin significantly increased the number of condensates formed in the presence of M1‐linked polyubiquitin (Figure 1E,F). P‐Optineurin incubated with M1‐ub_x_ chains assembled over time was also studied by a sedimentation assay, which uses centrifugation to separate the condensed and dilute phases. The distribution of proteins in both phases was analyzed by SDS‐PAGE and immunoblotting.^[^ ^56^, ^57^ ^]^ Without M1‐ub_x_, p‐Optineurin was exclusively found in the supernatant fraction (Figure 1G). In the presence of M1‐ub_x_, p‐Optineurin shifted into the pellet fraction together with M1‐ub_x_, confirming the formation of condensates. The pellet fraction mostly enriched high molecular weight M1‐ub_x_ species, suggesting that longer M1‐ub chains more efficiently promote p‐Optineurin condensation (Figure 1G). To analyze the effect of M1‐ub_x_ (formed after 120 min reaction time) in more detail, we quantified the number of condensates and their average volume at different time points after mixing p‐Optineurin with M1‐ub_x_. The number of condensates reached a plateau phase after ≈ 30 min, but the volume of the condensates increased over time (Figure 1H–J). In sum, Optineurin phosphorylated by TBK1 forms condensates in the presence of M1‐linked ubiquitin chains, which increase in size over time.

### 2.2 Phase Separation of Phosphorylated Optineurin is Preferentially Induced by M1‐Linked Ubiquitin Chains

**Figure 2:**
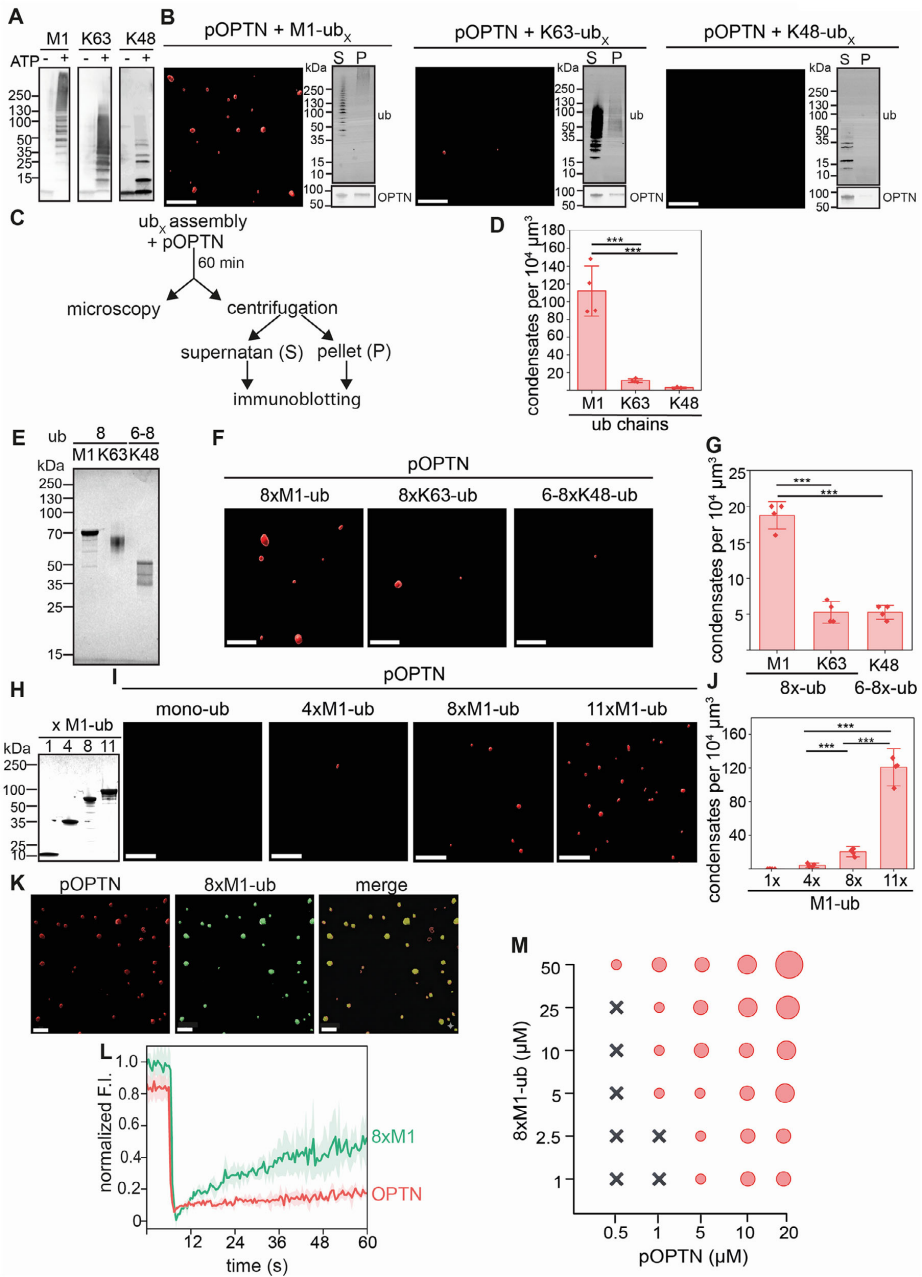
M1‐linked polyubiquitin chains are most effective in promoting condensation of phosphorylated Optineurin. A) In vitro enzymatic assembly of M1‐, K63‐ and K48‐linked ubiquitin chains using specific E2 ubiquitin‐conjugating enzymes and E3 ubiquitin ligases. The assembly reaction was initiated by ATP and stopped by acidification. The ubiquitin chains were analysed by immunoblotting using an anti‐ubiquitin antibody (P4D1). B,C) Analysis of pOPTN condensate formation in the presence of M1‐, K63‐ or K48‐linked ubiquitin chains by fluorescence microscopy (3D reconstruction) and sedimentation assays. M1‐linked polyubiquitin is more effective in inducing pOPTN condensation than K63‐ and K48‐linked polyubiquitin. Fluorescence microscopy and immunoblot analysis after sedimentation of condensates formed by pOPTN (5 µM) in the presence of M1‐, K63‐ or K48‐linked polyubiquitin chains (40 µM). pOPTN). Scale bar: 5 µm. D) Quantification of the number of pOPTN condensates corresponding to the conditions shown in B. Data represent the mean ± SD of four independent experiments. The statistical analysis was performed using a one‐Way ANOVA test with Tukey's multiple comparison test (***p ≤ 0.001). E) Immunoblot analysis of 8xM1‐ub, 8xK63‐ub and 6–8xK48‐ub generated in vitro (as described in a) and subsequently purified by cation exchange chromatography. F) 8xM1‐ub is more effective in inducing pOPTN condensation than 8xK63‐ub and 6–8xK48‐ub. Fluorescence microscopy (3D reconstruction) of pOPTN condensates formed in the presence of 8xM1‐ub, 8xK63‐ub or 6–8xK48‐ub chains (5 µM pOPTN, 25 µM polyubiquitin). Scale bar: 5 µm. G) Quantification of the number of pOPTN condensates corresponding to the conditions shown in F. Data represent the mean ± SD of four independent experiments. The statistical analysis was performed using one‐Way ANOVA test with Tukey's multiple comparison test (*** *p* ≤ 0.001). H,I) The number of pOPTN condensates increases with the length of M1‐linked ubiquitin chains generated in vitro (H). Fluorescence microscopy (3D reconstruction) of pOPTN condensates formed in the presence of mono‐ubiquitin (ub), 4xM1‐ub, 8xM1‐ub or 11xM1‐ub (5 µM pOPTN, 25 µM polyubiquitin). Scale bar: 5 µm. J) Quantification of the number of pOPTN condensates corresponding to the conditions shown in H. Data represent the mean ± SD of four independent experiments. The statistical analysis was performed using one‐Way ANOVA test with Tukey's multiple comparison test (*** *p* ≤ 0.001). K) Co‐condensation of pOPTN (5 µM) and 8xM1‐ubiquitin (25 µM) labelled with AF 488 shown by fluorescence imaging (3D reconstruction). Scale bar: 3 µm. L) FRAP analysis of pOPTN condensates formed in the presence of 8xM1‐ub labelled with AF 488 corresponding to K. Shown is the fluorescence recovery of pOPTN (red) and 8xM1‐ub (green). M) Concentration‐dependent phase separation of pOPTN and 8xM1‐ub. p‐Optineurin was incubated in presence of 8xM1‐ub for 60 min at the concentrations indicated and analysed by fluorescence microscopy. Black crosses: no phase separation; red circles: phase separation; the size of the circles correlates with the condensate volume.

To test whether ubiquitin‐induced phase separation of p‐Optineurin is specific for M1‐linked polyubiquitin, we also tested K63‐ and K48‐linked polyubiquitin. K63‐linked (K63‐ub_x_) and K48‐linked ubiquitin (K48‐ub_x_) chains were generated enzymatically in vitro by using UBE2V1/UBE2N and UBE2R1, respectively, as described previously.^[^ ^58^ ^]^ The enzymatic reaction was stopped, and the polyubiquitin chains were incubated with p‐Optineurin in the same buffer used for incubation with M1‐linked polyubiquitin. The formation of polyubiquitin chains was confirmed by immunoblotting (**Figure** **2**A). M1‐ub_x_ efficiently promoted phase separation of p‐Optineurin, whereas only a few condensates were observed in the presence of K63‐ub_x_ or K48‐ub_x_ (Figure 2B–D). Fluorescence microscopy was complemented by sedimentation assays, showing that p‐Optineurin most efficiently shifted to the pellet fraction together with M1‐ub_x_, and K63‐ub_x_, whereas neither p‐Optineurin nor K48‐ub_x_ was detected in the pellet fraction, confirming that condensate formation of p‐Optineurin was not induced by K48‐ub_x_ (Figure 2B,C).

Since the in vitro‐assembled polyubiquitin chains showed differences in their abundance and length spectrum, we purified octa‐ubiquitin chains linked by either M1 (8xM1‐ub) and K63 (8xK63‐ub), and a mixture of hexa‐ and octa‐K48 ubiquitin chains (6‐8xK48‐ub) (Figure 2E). Fluorescence microscopy revealed a similar trend with 8xM1‐ub being the most efficient linkage type in inducing p‐Optineurin condensates (Figure 2E–G). Next, we tested M1‐linked polyubiquitin chains of different lengths (4x, 8x, and 11xM1‐ub) and observed that undeca‐M1 ubiquitin (11xM1‐ub) had the strongest effect in promoting phase separation of p‐Optineurin compared to 4xM1‐ub and 8xM1‐ub (Figure 2H–J). We confirmed the co‐condensation of M1‐linked polyubiquitin by using 8xM1‐ub labelled with a green fluorophore (AF 488) and performed both fluorescence microscopy (Figure 2K). Fluorescence recovery after photobleaching (FRAP) was employed to analyze the material properties of the p‐Optineurin condensates. Whereas 8xM1‐ub showed a 40% recovery of fluorescence, pOPTN fluorescence did not recover, suggesting that the pOPTN condensates are solid‐like, similarly to condensates formed by the autophagy receptor p62 (Figure 2L).^[^ ^12^ ^]^ Treatment of the p‐Optineurin/8xM1‐ub condensates with 1,6% hexanediol did not induce dissolution of the condensates, confirming their solid nature (Figure S2A, Supporting Information), as also observed for p62. ^[^ ^13^ ^]^

To study the concentration‐dependent phase separation of p‐Optineurin, we used 8xM1‐ub. As indicated in the phase diagram, larger condensates formed with increasing concentrations of p‐Optineurin and 8xM1‐ub (Figure 2M). We then tested the influence of TBK1‐dependent phosphorylation sites on Optineurin phase separation. Recombinantly expressed single phosphomimic S177E, S473E or S513E Optineurin mutants were analysed in the presence of M1‐ub_x_. The S473E mutant (serine 473 located in the UBAN domain) exhibited a slight, yet statistically significant increase in the number of condensates compared to wild‐type non‐phosphorylated Optineurin (Figure S2B,C, Supporting Information). However, the number of condensates formed by S473E Optineurin did not reach the levels observed for p‐Optineurin (Figure 1F; Figure S2B,C, Supporting Information), indicating that not a single serine but several serines phosphorylated by TBK1 promote Optineurin phase separation. Moreover, the replacement of the three serines 177, 473, and 513 by alanines (S177A/S473A/S513A) did not abrogate Optineurin condensation after phosphorylation, suggesting that other serines or threonines can be phosphorylated by TBK1, which is in line with the phosphoproteomic analysis performed by Richter et al.^[^ ^30^ ^]^ (Figure S2D, Supporting Information). Taken together, although Optineurin can bind K63‐linked ubiquitin chains,^[^ ^31^, ^59^ ^]^ M1‐linked polyubiquitin was most efficient in inducing the phase separation of p‐Optineurin in a length‐ and concentration‐dependent manner.

### 2.3 The M1‐Ubiquitin‐Specific Deubiquitinase OTULIN Prevents and Reverses Optineurin Phase Separation

**Figure 3:**
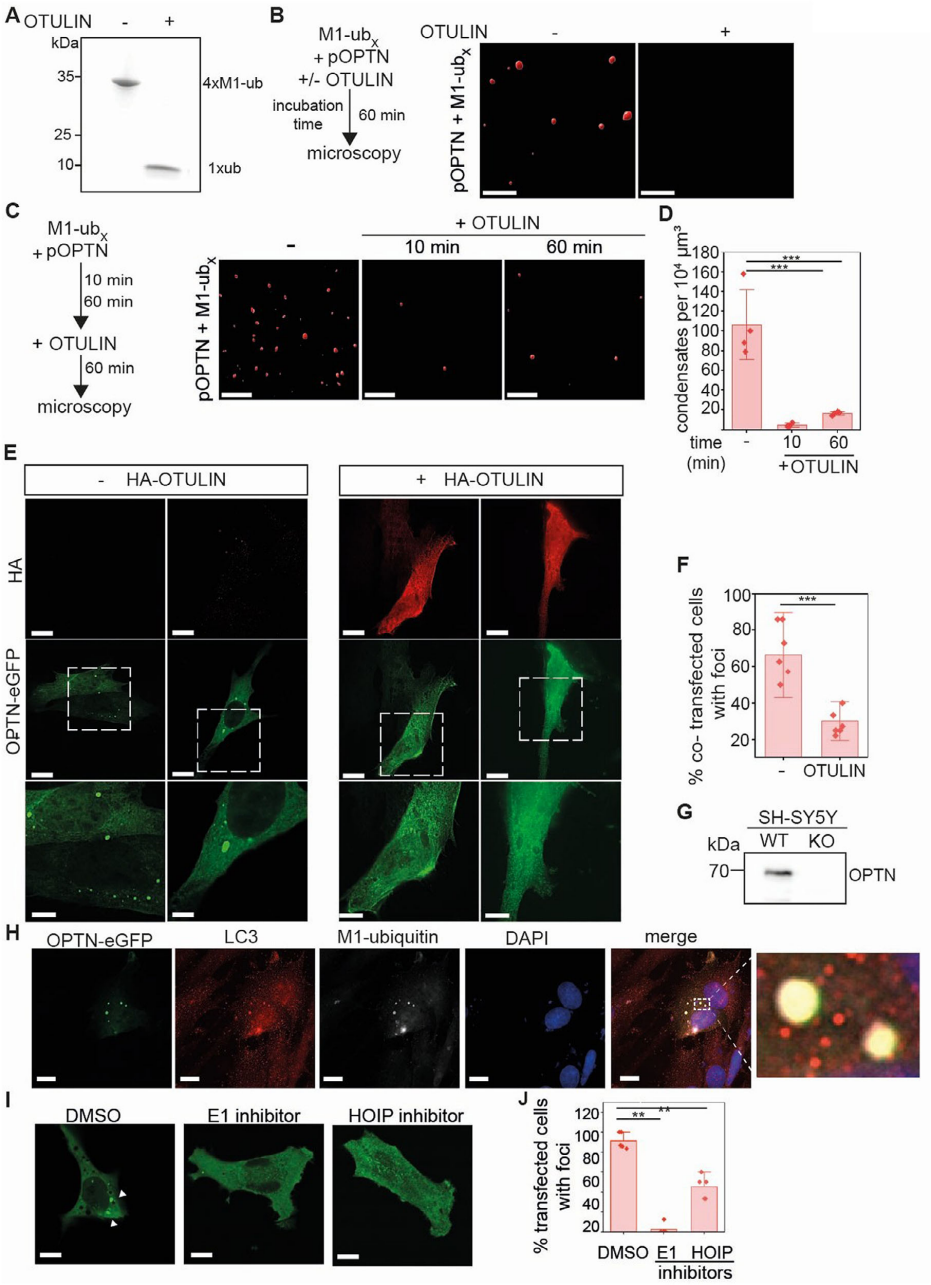
The M1‐ubiquitin‐specific deubiquitinase OTULIN prevents and reverses phase separation of phosphorylated Optineurin in vitro and in cells. A) Deubiquitylation activity of recombinant OTULIN. 4xM1‐ub (20 µM) was incubated with and without OTULIN (2 µM, 1 h at 37 °C) and analyzed by immunoblotting using an anti‐ubiquitin antibody (P4D1). B) OTULIN prevents the formation of pOPTN condensates. pOPTN (5 µM) and M1‐ub_x_ chains (40 µM) were incubated with or without OTULIN (5 µM) for 60 min followed by fluorescence microscopy (3D reconstruction). Scale bar: 5 µm. C) OTULIN reverses the formation of pOPTN condensates. pOPTN and M1‐ub_x_ were incubated for 10 or 60 min. Then OTULIN was added followed by fluorescence microscopy (3D reconstruction) after 60 min incubation in the presence of OTULIN. Scale bar: 5 µm. D) Quantification of the number of pOPTN condensates corresponding to the conditions shown in C. Data represent the mean ± SD of four independent experiments. The statistical analysis was performed using a Mann‐Whitney test (*** *p* ≤ 0.001). E) OTULIN suppresses the formation of Optineurin condensates in SH‐SY5Y cells. OPTN‐KO SH‐SY5Y cells generated by CRISPR/Cas9 were transiently transfected with Optineurin fused with eGFP (OPTN‐eGFP) and HA‐OTULIN or a control plasmid (‐ HA‐OTULIN) and analyzed by immunocytochemistry and fluorescence microscopy 24 h after transfection using an anti‐HA antibody. The lowest panel represents a zoom of the area marked by a square. Scale bar: 10 µm, zoom‐in scale bar: 5 µm. F) Percentage of transfected cells with pOPTN condensates corresponding to the conditions shown in E. The percentage of transfected cells (expressing OPTN‐eGFP and HA) showing at least one assembly with a diameter ≥ 0.5 µm was quantified. Three biological replicates with two fields of view each were analyzed. The bars indicate the mean ± SD and the data points of each replicate are presented as points. The statistical analysis was performed using a Mann‐Whitney test (*** *p* ≤ 0.001). G) Immunoblot analysis of wildtype (WT) and OPTN‐KO SH‐SY5Y cells, confirming the defective expression of Optineurin in OPTN‐KO SH‐SY5Y cells. An anti‐Optineurin antibody (HPA003279, Merck) was used for detection. H) Optineurin condensates formed in SH‐SY5Y cells co‐localize with M1‐linked ubiquitin chains. OPTN‐KO SH‐SY5Y cells transiently transfected with OPTN‐eGFP were analyzed by immunocytochemistry and fluorescence microscopy 24 h after transfection using the 1E3 anti‐M1‐ubiquitin and LC3 antibody. Scale bar: 10 µm. I) Inhibition of ubiquitylation or HOIP interferes with the formation of Optineurin condensates in SH‐SY5Y cells. OPTN‐KO SH‐SY5Y cells transiently transfected with OPTN‐eGFP were treated with the E1 ubiquitin‐activating enzyme inhibitor TAK243 (1 µM, 24 h) or the HOIP inhibitor HOIPIN‐8 (30 µM, 24 h) 4 h after transfection and then analyzed by fluorescence microscopy. Scale bar: 10 µm. J) Percentage of transfected cells with Optineurin condensates corresponding to the conditions shown in I. Cells expressing OPTN‐eGFP were classified as positive when they showed at least one assembly ≥ 0.5 µm in diameter. Five areas of view each from three biological samples were analyzed. The statistical analysis was performed using a Mann‐Whitney test (** *p* ≤ 0.0025).

The formation of Optineurin condensates induced by phosphorylation and binding to M1‐linked ubiquitin chains points toward a tight regulation of this process. We therefore wondered whether the disassembly of M1‐linked chains is a regulatory mechanism to influence p‐Optineurin condensation. OTULIN is the only deubiquitinase that exclusively hydrolyzes M1‐linked polyubiquitin.^[^ ^60^, ^61^, ^62^, ^63^, ^64^ ^]^ We purified human OTULIN expressed in *E. coli* and confirmed its activity to hydrolyze 4xM1‐ub chains in an in vitro deubiquitylation assay (**Figure** **3**A). When OTULIN was mixed with p‐Optineurin and M1‐ub_x_ chains and incubated for 60 min, OTULIN completely prevented the formation of condensates (Figure 3B). Even adding OTULIN 10 min or 60 min after starting the incubation of p‐Optineurin and M1‐ub_x_ significantly reduced the number of p‐Optineurin condensates, indicating that hydrolysis of M1‐linked polyubiquitin by OTULIN results in the disassembly of p‐Optineurin condensates (Figure 3C,D). We also tested whether OTULIN influences the formation of Optineurin condensates in cells. We generated Optineurin CRISPR/Cas9 knockout (OPTN‐KO) SH‐SY5Y cells, allowing us to express tagged Optineurin or Optineurin variants in an Optineurin‐deficient genetic background (Figure 3E–G). The ectopic expression of Optineurin fused to enhaced green fluorescent protein (eGFP) in these cells resulted in the formation of Optineurin foci in ≈ 69% of transfected cells without any additional stimulus (Figure 3E,F). When OTULIN was co‐expressed, the fraction of cells containing Optineurin foci was reduced to 30% (Figure 3E,F). The Optineurin foci formed in SH‐SY5Y cells also stained positive for phospho‐S177 Optineurin, M1‐ubiquitin, and LC3 (Figure 3H; Figure S3A, Supporting Information). Moreover, the increased expression of HOIP in wildtype SH‐SY5Y cell promoted the formation of Optineurin, TBK1, phospho‐TBK1, M1‐ub positive foci that co‐localize with TBK1 (Figure S3B, Supporting Information), as observed for poly(I:C)‐treated cells. ^[^ ^65^ ^]^ To confirm a role of ubiquitin and in particular of M1‐linked polyubiquitin, we treated OPTN‐KO cells expressing Optineurin‐eGFP with either the E1 ubiquitin‐activating enzyme inhibitor TAK‐243 or the HOIP inhibitor HOIPIN‐8.^[^ ^66^ ^]^ The formation of Optineurin foci was abrogated by the E1 inhibitor and reduced by ≈ 50% in the presence of the HOIP inhibitor (Figure 3I,J), similarly to the effect of increased OTULIN expression. In conclusion, the inhibition of M1‐ubiquitylation and the disassembly of M1‐linked ubiquitin chains interferes with Optineurin condensation and foci formation in vitro and in cells.

### 2.4 Condensation of Phosphorylated Optineurin is Disrupted by Pathogenic Mutations Within the UBAN Domain

**Figure 4:**
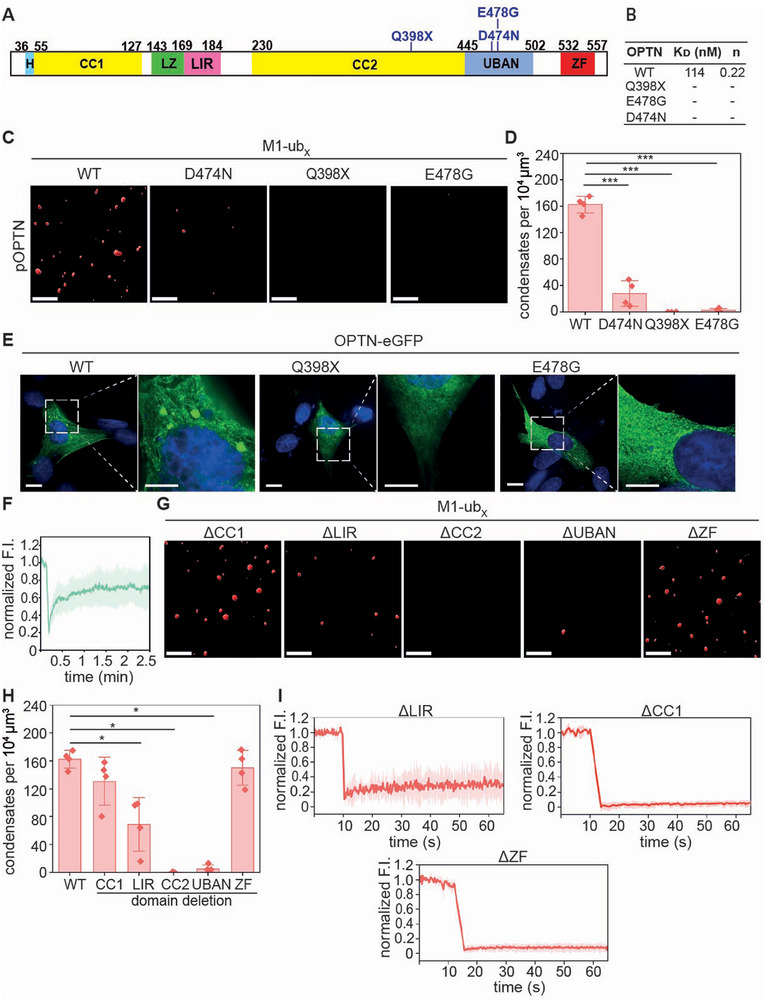
Ubiquitin binding‐deficient pathogenic Optineurin variants do not form condensates in the presence of M1‐linked polyubiquitin. A) Domain structure of Optineurin (OPTN) with the mutations analyzed indicated in blue. B) WT and mutated OPTN binding to 2xM1‐ub measured by isothermal titration calorimetry. C) OPTN mutants affecting the UBAN domain do not form condensates in the presence of M1‐ub_x_ ubiquitin. Fluorescence microscopy (3D reconstruction) of condensates formed by p‐OPTN variants (5 µM) in the presence of M1‐ub_x_ (40 µM). Scale bar: 5 µm. D) Quantification of the number of pOPTN condensates corresponding to the conditions shown in C. Data represent the mean ± SD of four independent experiments. The statistical analysis was performed using a Mann‐Whitney test (*** *p* ≤ 0.001). E) OPTN UBAN mutants do not form condensates in cells. OPTN‐KO SH‐SY5Y cells were transiently transfected with either wildtype (WT) OPTN‐eGFP, Q398X OPTN‐eGFP or E478G OPTN‐eGFP and analyzed by fluorescence microscopy 24 h after transfection. Representative images from three biological replicates are shown. 6 to 10 cells per condition were analyzed for each biological replicate. Scale bar: 10 µm, zoom in scale bar: 5 µm. F) FRAP analysis of wildtype OPTN‐eGFP foci in cells. Average normalized fluorescence intensity of three AOIs was plotted over time. The curve is presented as means ± SD, n = 8. G) The UBAN and CC2 domains are required for the condensation of pOPTN in the presence of M1‐ub_x_. The OPTN variants indicated were purified and phosphorylated by TBK1. Fluorescence microscopy (3D reconstruction) was performed after incubation of pOPTN (5 µM) with M1‐ub_x_ (40 µM) for 60 min. Scale bar: 5 µm. H) Quantification of the number of pOPTN condensates corresponding to the conditions shown in F. Data represent the mean ± SD of four independent experiments. The statistical analysis was performed using a Mann‐Whitney test (* *p* ≤ 0.05). I) FRAP analysis of ΔLIR, ΔZF, and ΔCC1 pOPTN deletion mutants. Average normalized fluorescence intensity of three AOIs was plotted over time.

A missense (E478G) and a nonsense mutation (Q398X) in the *OPTN* gene affecting the UBAN domain are linked to ALS.^[^ ^38^, ^67^ ^]^ The E478G variant is impaired in its binding to M1‐linked polyubiquitin^[^ ^24^ ^]^ and the Q398X variant lacks the UBAN domain, the ZF domain and the C‐terminal part of the CC2 domain (**Figure** **4**A). In addition to these variants, we expressed and purified D474N Optineurin, a UBAN variant showing decreased binding to polyubiquitin.^[^ ^59^, ^68^ ^]^ All purified Optineurin variants were phosphorylated by TBK1 and labeled with Cy5 (Figure S4A,C, Supporting Information). First, we analysed their ability to bind to 2xM1‐ub in vitro by isothermal titration. The dissociation constant (K_D_)_)_ for the interaction between wildtype Optineurin and 2xM1‐ub was 0.11 µM, whereas no binding of the three Optineurin variants to 2xM1‐ub was observed (Figure 4B; Figure S4D, Supporting Information). We then tested condensation of the phosphorylated Optineurin variants in the presence of M1‐ub_x_ chains. Q398X and E478G p‐Optineurin did not form condensates under these conditions and only a few condensates were observed for D474N p‐Optineurin (Figure 4C,D). To study foci formation in a cellular context, we expressed the Optineurin variants E478G or Q398X fused to eGFP in OPTN‐KO SH‐SY5Y cells. In contrast to cells expressing wildtype Optineurin, no GFP‐positive foci were observed in cells expressing E478G or Q398X Optineurin, confirming the relevance of the UBAN domain for Optineurin foci formation in a cellular context (Figure 4E). The FRAP analysis of foci formed by wildtype Optineurin in cells showed a recovery of ≈ 80%, suggesting that the Optineurin condensates formed in vitro and in cells show different material properties (Figure 4F), which has also been described for p62.^[^ ^13^ ^]^

We then tested if other domains predicted to contain IDRs, such as the CC1, LIR, CC2, and ZF domains (Figure 1B), influence p‐Optineurin condensation. The purified domain deletion variants ΔCC1, ΔLIR, ΔCC2, ΔUBAN and ΔZF Optineurin variants were phosphorylated by TBK1 and labeled with Cy5 (Figure S4B,C, Supporting Information). Interestingly, the deletion of both the UBAN and CC2 domain prevented the formation of p‐Optineurin condensates in the presence of M1‐ub_x_, and the deletion of the LIR domain reduced the number of p‐Optineurin condensates (Figure 4G,H). FRAP experiments showed that the condensates formed by ΔCC1, ΔZF and ΔLIR p‐Optineurin are quite undynamic, similarly to wildtype p‐Optineurin (Figure 4I). Thus, an intact UBAN domain and the CC2 domain are required for p‐Optineurin phase separation, whereas deletion of the C‐terminal CC1domain or the N‐terminal ZF domain did not significantly affect condensate formation.

Optineurin plays a role in the quality control and autophagic clearance of protein aggregates. ^[^ ^23^, ^26^, ^31^, ^67^, ^69^ ^]^ We therefore tested the recruitment of pathogenic Optineurin mutants in a cellular model of protein aggregation. We expressed Huntingtin exon 1 with an extended poly‐glutamine stretch (Htt‐Q97‐mScarlet) in SH‐SY5Y cells and observed co‐localization of endogenous Optineurin, p‐Optineurin, TKB1 and LC3 (Figure S5A, Supporting Information), consistent with previous findings.^[^ ^26^ ^]^ When Htt‐Q97‐expressing SH‐SY5Y cells were treated with the TBK1 inhibitor GSK8612, the recruitment of endogenous Optineurin was decreased although M1‐ubiquitin chains were present at the Htt‐Q97 aggregates, suggesting that TBK1‐induced phosphorylation promotes the binding of Optineurin to Htt‐Q97 aggregates decorated with M1‐ubiquitin (Figure S5B,C, Supporting Information). We used OPTN KO SH‐SY5Y cells expressing Htt‐Q97 to compare the behavior of wildtype and pathogenic Optineurin mutants. Wild‐type Optineurin formed foci in close proximity to Htt‐Q97 aggregates. Within these foci, optineurin showed 20–30% recovery within 60 s after photobleaching. (Figure S5D, Supporting Information). E478G and Q398X Optineurin did not form foci. However, both mutants colocalized with Htt‐Q97 aggregates and were completely undynamic, suggesting co‐aggregation with Htt‐Q97 (Figure S5D, Supporting Information).

### 2.5 Soluble and Membrane‐Bound LC3 Co‐condensates with Phosphorylated Optineurin

**Figure 5:**
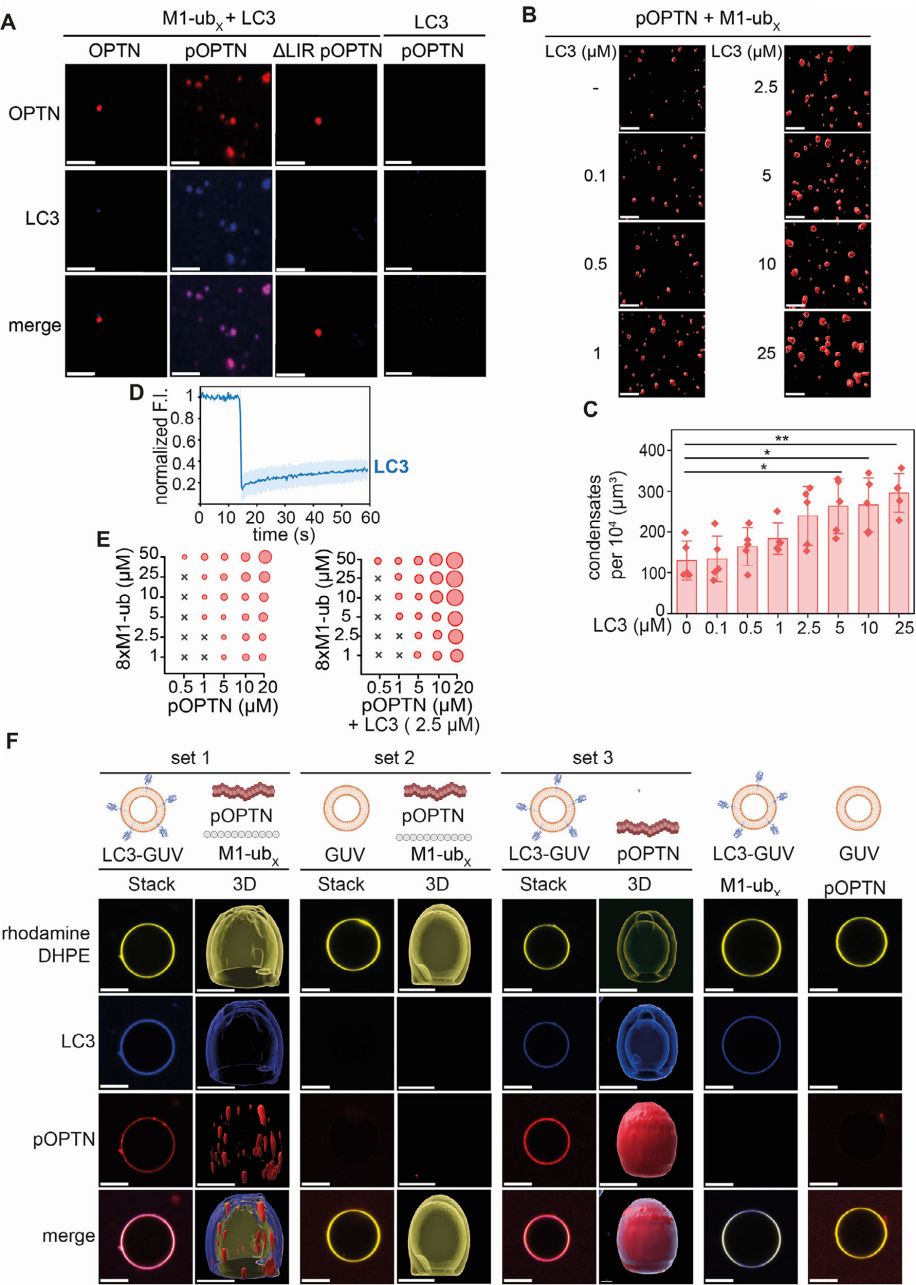
Soluble and membrane‐bound LC3 co‐partitions into condensates formed by phosphorylated Optineurin. A) Soluble LC3 co‐condensates with pOPTN and M1‐ub_x_. Recombinant LC3 (5 µM) was incubated with pOPTN or ΔLIR pOPTN (5 µM) in the presence or absence of M1‐ub_x_ (40 µM) and analyzed by fluorescence microscopy after 60 min. Scale bar: 5 µm. B) LC3 increases the number and volume of pOPTN/M1‐ub_x_ condensates in a concentration‐dependent manner. Purified LC3 was incubated with pOPTN/M1‐ub_x_ (5 µM/40 µM) and fluorescence microscopy (3D reconstruction) was performed after 60 min. Scale bar: 5 µm. C) Quantification of the number of pOPTN condensates corresponding to the conditions shown in B. Data represent the mean ± SD of f independent experiments. The statistical analysis was performed using a Mann‐Whitney test (* *p* ≤ 0.05, **p ≤ 0.01). D) FRAP analysis of LC3 in condensates formed by pOPTN/M1‐ub_x_ corresponding to the conditions shown in A. Average normalized fluorescence intensity of three AOIs was plotted over time. E) Concentration‐dependent phase separation of pOPTN and 8xM1‐ub in the presence or absence of soluble LC3. pOPTN was incubated with 8×M1‐ub ± LC3 (2.5 µM) for 60 min and then analyzed by fluorescence microscopy. Black crosses: no phase separation; red circles: phase separation; the size of the circles correlates to the condensate volume. F) pOPTN is recruited to LC3‐coated GUVs but only forms condensates at GUVs in the presence M1‐ub_x_. LC3‐coated (2.5 µM) or uncoated GUVs were incubated with proteins indicated (pOPTN: 5 µM; M1‐ub_x_: 40 µM) and confocal fluorescence microscopy was performed after 60 min. Shown are representative images from three independent experiments. Scale bar: 5 µm; scale bar 3D reconstruction: 3 µm.

Phosphorylation of Optineurin by TBK1 also increases its binding to LC3 ^[^ ^19^, ^30^ ^]^ Since the LIR domain influenced phase separation of p‐Optineurin, we tested whether LC3 co‐condensates with p‐Optineurin. Purified LC3 fused to a cyan fluorescent protein derived from *Aequorea victoria* (CFP) was incubated with either unphosphorylated Optineurin, p‐Optineurin, or p‐Optineurin lacking the LIR domain (ΔLIR) in the presence of M1‐ub_x_ and the formation of condensates was assessed after 60 min by fluorescence microscopy. LC3 formed co‐condensates with p‐Optineurin, but not with non‐phosphorylated Optineurin or with p‐Optineurin lacking the LIR domain (**Figure** **5**A). Of note, co‐condensation of LC3 and p‐Optineurin did not occur in the absence of M1‐ub_x_ (Figure 5A), indicating that M1‐polyubiquitin chains are required for co‐condensation, although LC3 interacts with Optineurin in the absence of ubiquitin chains (Figure 5F). With increasing concentrations of LC3 (0.1 to 25 µM), the number and average volume of p‐Optineurin/M1‐ub_x_ condensates increased (Figure 5B,C; Figure S6, Supporting Information). LC3 showed ≈ 20% recovery of fluorescence after photobleaching, suggesting a reduced dynamicity within the p‐Optineurin condensates (Figure 5D). We then tested the concentration‐dependent phase separation of p‐Optineurin and M1‐ub_x_ in the presence of LC3 (2.5 µM). The resulting phase diagrams showed that LC3 increased the volume of p‐Optineurin co‐condensates, but did not shift the phase separation of p‐Optineurin to a lower concentration (Figure 5E).

In selective autophagy, p‐Optineurin interacts with lipidated LC3 bound to the phagophore membrane. To mimic a membrane environment, we generated giant unilamellar vesicles (GUVs; 97.85% POPC, 2% DGS NTA‐Ni, 0.15% rhodamine DHPE) and anchored CFP‐LC3 to the membrane by its 6xHis tag. The LC3‐coated GUVs were incubated with p‐Optineurin/M1‐ub_x_ condensates and confocal fluorescence microscopy was performed after 60 min. The 3D reconstruction of the Z stacks showed the presence of p‐Optineurin/M1‐ub_x_ condensates at GUVs coated with LC3 (Figure 5F, set 1). Without LC3 coating, p‐Optineurin/M1‐ub_x_ condensates did not bind to GUVs (Figure 5F, set 2). When p‐Optineurin was incubated with LC3‐coated GUVs in the absence of M1‐ub_x_, uniform binding of p‐Optineurin to the GUVs was observed but no formation of condensates (Figure 5F, set 3), confirming our previous results that LC3 and p‐Optineurin do not form condensates in the absence of M1‐linked polyubiquitin (Figure 5A). These experiments revealed that LC3 co‐condensates with p‐Optineurin and M1‐linked ubiquitin chains at membranes, suggesting that LC3 could recruit p‐Optineurin/M1‐ub_x_ condensates formed at the cargo interface to the phagophore membrane.

### 2.6 Phosphorylated Optineurin Forms Filaments that Interact with LC3 Anchored to Unilamellar Vesicles

**Figure 6:**
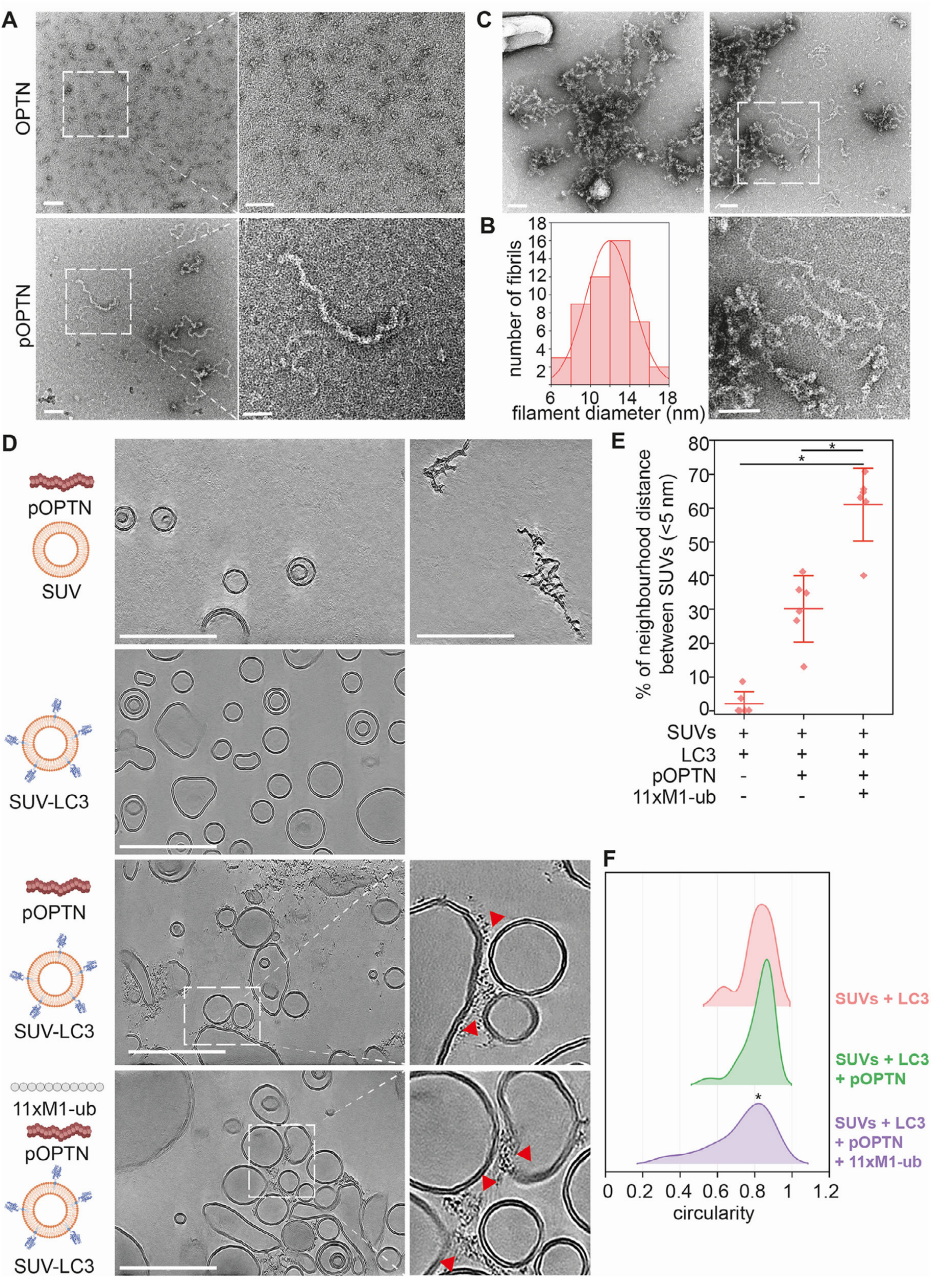
Phosphorylation of Optineurin by TBK1 promotes its self‐association into flexible filaments. A) pOPTN forms filaments. OPTN and pOPTN (1.15 µM in 20 mM Tris‐HCl, 250 mM NaCl, 2 mM TCEP) were analyzed by negative staining electron microscopy. Scale bar: 100 nm, zoom‐in scale bar: 50 nm. B) Diameter distribution of pOPTN filaments analyzed by negative staining electron microscopy. The quantification yields 12 ± 2.4 nm (average ± SD, n = 25) based on five different images. C) pOPTN filaments form large condensates in the presence of 11xM1‐ub. pOPTN (5 µM) was mixed with 11xM1‐ub (15 µM) and after 60 min analyzed by negative staining electron microscopy. Scale bar: 100 nm left; zoom‐in scale bar: 50 nm. D) pOPTN binds to LC3‐coated SUVs. pOPTN was mixed with LC3‐coupled or uncoupled SUVs in the absence or presence of 11xM1‐ub. SUVs were prepared at a final concentration of 3 mg mL^−1^ and incubated for 1 h with CFP‐LC3‐His (5 µM). Then, p‐OPTN (5 µM) and 11xM1‐ub (15 µM) were added and incubated for 1 h. The samples were analyzed by cryo‐ET and denoised tomogram slices are shown. Red arrowheads in the insets indicate the presence of protein next to the SUVs. Scale bar: 200 nm. E) pOPTN and M1‐linked polyubiquitin promote the clustering of LC3‐coated SUVs. Shown is the percentage of neighborhood distance (< 5 nm) between SUVs. Six tomograms per condition were quantified. The statistical analysis was performed using a Kruskal‐Wallis test (** *p* ≤0.01, * *p* ≤0.05). F) pOPTN and M1‐linked polyubiquitin alter the shape of SUVs assessed by a decrease in the circularity of LC3‐coated SUVs. The normalized distribution plot shows the average of three tomograms per condition. SUVs + LC3: n = 17; SUVs + LC3 + pOPTN: n = 51; SUVs + LC3 + pOPTN + 11xM1‐ub: n = 59. The statistical analysis was performed using a Kruskal‐Wallis test (*p = 0.048).

The specific property of p‐Optineurin to form condensates, which was not observed for non‐phosphorylated Optineurin, suggests a conformational change after phosphorylation that promotes phase separation of Optineurin in the presence of M1‐ubiquitin chains. To address this possibility, negative staining electron microscopy was performed using uranyl acetate for both phosphorylated and non‐phosphorylated recombinant Optineurin. Inhomogeneous smaller and medium‐sized particles deposited on the grid were observed for non‐phosphorylated Optineurin. In contrast, p‐Optineurin formed extended ribbon‐like filaments that can be twisted with an average diameter of 12 ± 2.4 nm (**Figure** **6**A,B). To investigate if Optineurin condensation is mediated by filamentous structures, we incubated p‐Optineurin and 11xM1‐ub for 1 h followed by negative staining electron microscopy. We observed µm‐sized agglomerated structures surrounded by particles and filaments that form clusters, compatible with the condensates previously observed by fluorescence microscopy (Figure 6C). We then tested if p‐Optineurin filaments can interact with membrane‐anchored LC3 by cryogenic electron tomography (cryo‐ET) visualization. Small unilamellar vesicles (SUVs) were prepared with a NTA anchor lipid (98% POPC, 2% DGS‐NTA‐Ni) to allow anchoring of CFP‐LC3 to the membrane via its 6×His tag. The LC3‐coated SUVs were then incubated with p‐Optineurin in the presence or absence of 11xM1‐ub for 60 min. As controls, LC3‐conjugated SUVs (CFP‐LC3‐SUVs) in the absence of p‐Optineurin and SUVs without CFP‐LC3 coating in the presence of p‐Optineurin were analyzed. Only naked liposomes and separated p‐Optineurin condensates were observed in the control tomograms (Figure 6D). In the samples containing p‐Optineurin and CFP‐LC3‐SUVs, proteinaceous densities compatible with the observed filaments emerged on the lipid surface, often linking multiple SUVs. Furthermore, additional SUV clustering and vesicle shape deformation were observed in tomographic slices when 11xM1‐ub was co‐incubated with p‐Optineurin. Measurements of the distances between SUVs in the neighborhood confirmed an increased tendency to cluster in the presence of 11xM1‐ub. (Figure 6E). When assessing the circularity of the SUVs in the respective samples, we noted a significant decrease in circularity when 11xM1‐ub was added (Figure 6F). Taken together, phosphorylation of Optineurin induces the formation of elongated filamentous condensates that bring M1‐linked ubiquitin chains and membrane‐anchored LC3 into close proximity. This process may promote cargo sequestration and engulfment at the expanding phagophore.

## 3 Discussion

**Figure 7:**
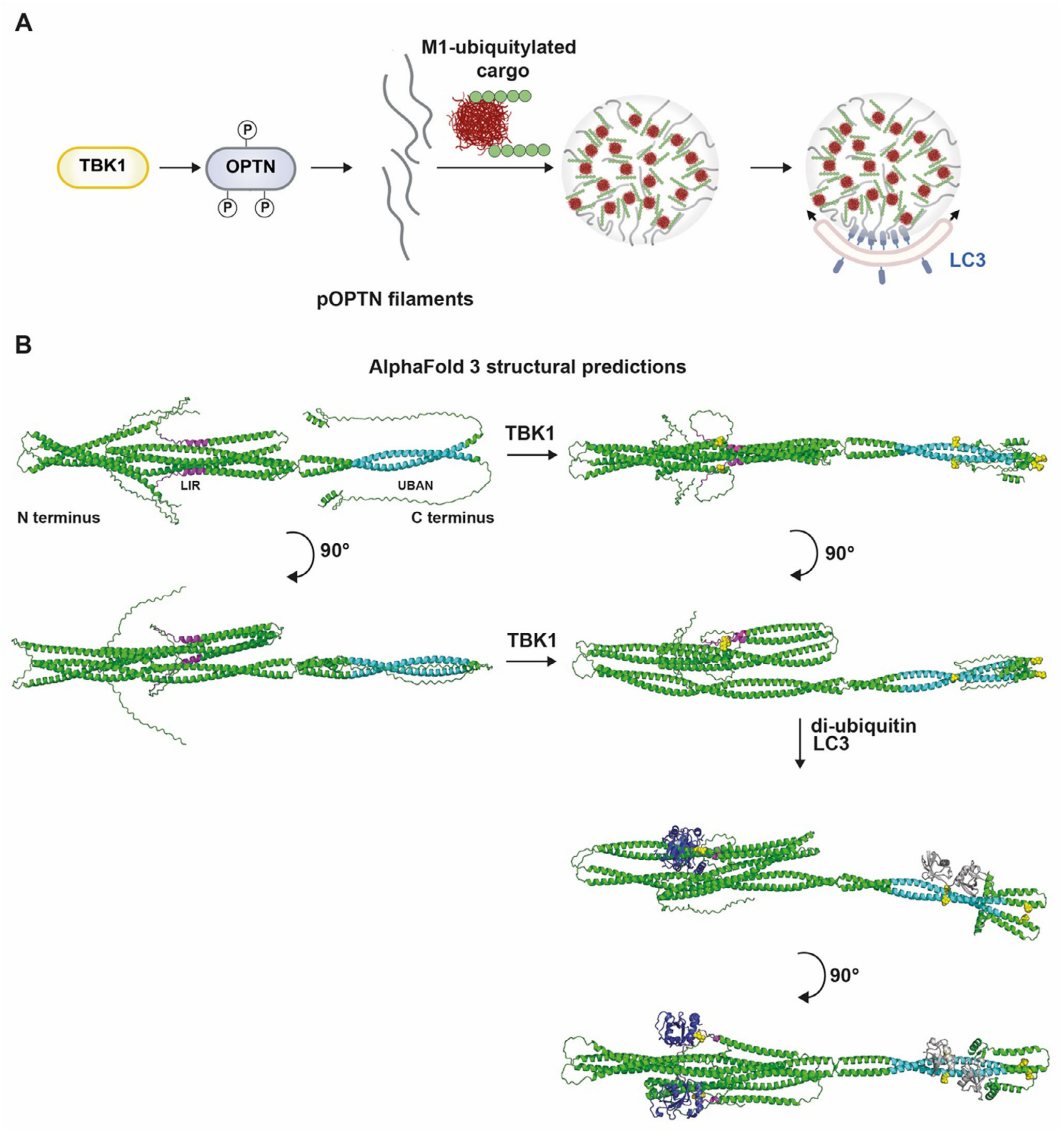
Model of Optineurin folding and filament formation and condensation in selective autophagy. A) Phosphorylation of Optineurin by TBK1 induces the formation of filaments that condensate upon binding to linear polyubiquitin. Membrane‐anchored LC3 co‐partitions into these condensates, suggesting that phase separation of filamentous Optineurin with ubiquitylated cargo, such as protein aggregates, promotes the sequestration of cargo and its subsequent alignment with LC3‐positive nascent autophagosomes. B) Prediction of the dimeric OPTN structure by AlphaFold 3 and its changes upon TBK1‐mediated phosphorylation of serine 177 and 473, binding of M1‐linked di‐ubiquitin, and LC3. LIR domain (magenta), UBAN domain (cyan), phospho‐serines 177, 473 (yellow), di‐ubiquitin (grey), and LC3 (blue).

In this study, we have shown that phosphorylated Optineurin forms filaments and undergoes phase separation upon binding to M1‐linked ubiquitin chains. Phosphorylation of Optineurin by TBK1 is a prerequisite for both Optineurin filament formation and its phase separation upon binding to polyubiquitin. Cryo‐ET showed that clusters of phosphorylated Optineurin (p‐Optineurin) filaments and M1‐linked polyubiquitin align LC3‐coated unilamellar vesicles and can locally deform the membrane surface. This is consistent with a model in which p‐Optineurin and M1‐linked ubiquitin chains by condensation promote cargo nucleation followed by LC3 co‐condensation at the growing autophagosomal membrane to provide directionality toward cargo engulfment (**Figure** **7**A).

According to the AlphaFold 3 prediction,^[^ ^70^ ^]^ Optineurin exhibits an extended conformation due to the coiled‐coil domains, with the LIR and UBAN domains spatially distant from one another (Figure 7B; Figure S7A, Supporting Information). Upon phosphorylation, Optineurin is predicted to adopt a conformation that is more accessible for both di‐ubiquitin and LC3 (Figure 7B; Figure S7B,C, Supporting Information). In addition, the negative charges of phosphorylated serines may contribute to electrostatic interactions at the binding interfaces in analogy to the observed acidic basic interfaces of the Phox and Bem1p domain (PB1) from the autophagy receptor p62.^[^ ^16^ ^]^

It was previously shown that phosphorylation by TBK1 increases the affinity of Optineurin to polyubiquitin chains.^[^ ^30^, ^31^, ^32^, ^33^ ^]^ Binding studies of UBAN domains and di‐ or tetra‐M1‐linked ubiquitin were mostly performed with isolated UBAN domains and indicated that interactions between charged residues play a role in the increased affinity of the phosphorylated UBAN domain and ubiquitin chains. Our study suggests that filament formation of full‐length Optineurin contributes to the binding of p‐Optineurin to polyubiquitin by increasing multivalency through oligomerization and possibly by increasing accessibility of the UBAN domain upon TBK1‐mediated phosphorylation.

Compared to K63‐ and K48‐linked polyubiquitin, M1‐linked ubiquitin chains most potently induced phase separation of p‐Optineurin with an increase in the number of condensates with increasing chain length. Confirming a critical role of M1‐linked polyubiquitin in inducing p‐Optineurin condensation, the M1‐ubiquitin linkage‐specific deubiquitinase OTULIN was able to prevent and partially reverse p‐Optineurin phase separation in vitro. Consistent with the in vitro results, increased expression of OTULIN in cultured cells or treatment of the cells with the HOIP inhibitor HOIPIN‐8 strongly decreased the formation of Optineurin condensates. These findings point toward a fine‐tuned regulation of Optineurin condensation by both TBK1‐mediated phosphorylation and the M1‐ubiquitylation machinery, which most probably is relevant to the role of Optineurin in both signaling and selective autophagy. M1‐ubiquitin chains regulate various receptor‐mediated immune signaling pathways resulting in NF‐κB activation.^[^ ^71^, ^72^, ^73^, ^74^, ^75^ ^]^ We and others found that binding of NEMO to M1‐ and K63‐linked ubiquitin chains induces the formation of signaling‐competent condensates in cells upon TNF or IL‐1β receptor activation.^[^ ^44^, ^76^ ^]^ Optineurin has been reported to antagonize the activation of NF‐κB, and competition with NEMO for polyubiquitin binding is one of the underlying mechanisms.^[^ ^68^, ^77^ ^]^ In line with this notion, Optineurin foci induced by poly(I:C) treatment of cells to mimic viral infection sequestered NF‐κB pathway components, including LUBAC.^[^ ^65^ ^]^ As a selective cargo receptor, p‐Optineurin primarily binds to M1‐linked and K63‐linked ubiquitin chains, which are assembled at intracellular bacteria and protein aggregates.^[^ ^19^, ^20^, ^22^, ^23^, ^24^, ^31^, ^42^, ^43^, ^78^ ^]^ Although M1‐ubiquitin chains do not play a major role in PINK1/Parkin‐dependent mitophagy,^[^ ^79^, ^80^, ^81^ ^]^ the high local concentration of K63‐linked polyubiquitin on depolarized mitochondria is obviously sufficient to promote p‐Optineurin condensation.^[^ ^30^, ^33^, ^39^, ^40^, ^82^, ^83^ ^]^

Another interesting observation of our study was that the deletion of the LIR motif reduced the number of p‐Optineurin condensates. We therefore studied a possible influence of LC3 on p‐Optineurin condensation. Soluble LC3 or LC3 coupled to unilamellar vesicles (SUVs and GUVs) interacted with p‐Optineurin, but did not induce p‐Optineurin condensation in the absence of M1‐linked polyubiquitin. However, LC3 partitioned into p‐Optineurin/M1‐linked polyubiquitin condensates and increased their size and number in a concentration‐dependent manner. An important step in selective autophagy is the alignment of ubiquitylated cargo with the expanding LC3‐decorated phagophore, allowing cargo segregation and engulfment. Our ultrastructural analysis indicated that p‐Optineurin in contrast to non‐phosphorylated Optineurin forms filaments, which can act as scaffolds for nucleating ubiquitylated cargo and aligning cargo by co‐condensation with LC3 at the phagophore, suggesting that condensation also promotes processivity of events in selective autophagy.

The ALS‐linked Optineurin variants E478G and Q398X, which are defective in polyubiquitin binding through the UBAN domain, did not form condensates in vitro or foci in cellular models. It has been reported before that Optineurin UBAN variants, such as E478G or ΔUBAN, are recruited to protein aggregates when exogenously expressed in an Optineurin wildtype genetic background.^[^ ^23^, ^26^ ^]^ This was explained by the fact that mutant Optineurin can oligomerize with endogenous wildtype Optineurin colocalizing with protein aggregates.^[^ ^23^ ^]^ As an alternative, a ubiquitin‐independent recruitment mechanism has been proposed.^[^ ^26^ ^]^ We used OPTN KO SH‐SY5Y cells to study the recruitment of the pathogenic Optineurin variants E478G and Q398X to Htt‐Q97 aggregates in an Optineurin‐deficient background in order to avoid an impact of endogenous wildtype Optineurin. In line with previous studies, the pathogenic mutants co‐localized with Htt‐Q97 aggregates,^[^ ^26^, ^31^ ^]^ which most probably can be explained by the presence of TBK1 at the aggregates. However, in contrast to wildtype Optineurin, the E478G and Q398X variants did not form foci at Htt‐Q97 aggregates. This could explain why these Optineurin variants although present at protein aggregates are not effective in promoting aggrephagy.^[^ ^31^ ^]^ Local enrichment of Optineurin in condensates and co‐condensation with LC3 at the growing autophagophore could be required for the efficient removal of protein aggregates by autophagosomes. Supporting this scenario, the autophagy receptor p62, also forms filaments and condensates with ubiquitylated cargo to drive autophagosomal degradation.^[^ ^12^, ^16^, ^84^ ^]^ A neuropathological analysis of three ALS patients with the E478G or Q389X mutation revealed TDP‐43 pathology, as well as tau and α‐synuclein aggregation. Interestingly, a widespread mixed brain proteinopathy including α‐synuclein, tau and TDP‐43 pathology was also detected in a patient with a mutation in the NEMO gene, which disrupts NEMO binding to M1‐linked polyubiquitin.^[^ ^43^ ^]^ NEMO is recruited to intracellular protein aggregates, where it promotes the co‐condensation of p62 and polyubiquitin, thereby facilitating their autophagosomal removal. Therefore, despite lacking a LIR domain, NEMO can act as an indirect autophagy receptor.

It is noteworthy that protein condensation upon binding to ubiquitin chains is not limited to autophagy but rather is a general phenomenon of protein quality control. For instance, phase separation of the proteasomal shuttling factors ubiquilin 2 (UBQLN2) and UV excision repair protein RAD23 homolog B (RAD23B) is regulated by their binding to ubiquitylated proteins.^[^ ^85^, ^86^, ^87^, ^88^, ^89^, ^90^ ^]^ During protein quality control, protein ubiquitylation and recruitment of quality control components to ubiquitylated proteins already occurs at the stage of misfolded soluble or oligomeric proteins, not only at the final aggregates.^[^ ^26^, ^43^, ^78^ ^]^ Thus, ubiquitylation determines the fate of misfolded cargo at different stages of the aggregation process, depending on multiple parameters of the complex ubiquitin code, such as linkage type, chain length, and heterotypic chain formation.^[^ ^88^, ^91^, ^92^ ^]^ Further studies are needed to address the determinants of pathway choices in protein quality control in more detail and whether filament formation is a more common mechanism by which cargo receptors promote autophagosomal degradation.

## 4 Experimental Section

### DNA Constructs

The following constructs were previously described: pGST2‐GST‐TEV‐OPTN, pFastBac‐Dual‐GST‐TEV‐TBK1, pGEX‐M1‐linked tetra‐ubiquitin.^[^ ^34^, ^93^, ^94^ ^]^ The GST‐Optineurin variants, GST‐di‐ubiquitin and CFP‐LC3‐His, were generated using the Q5 mutagenesis kit (E0554S, New England Biolabs, see primers Table 1). UBE2N was cloned into a pGEX plasmid and HA‐OTULIN and Htt‐Q97‐mScarlet were cloned pcDNA 3.1 plasmids, respectively. The following plasmids were obtained from Addgene: pEGFP‐N1‐OPTN‐WT (Addgene plasmid number: 27052), pEGFP‐N1‐OPTN‐Q398X(Addgene plasmid number: 68849), pEGFP‐N1‐OPTN‐E478G (Addgene number: 68848), pET28b His‐mouse UBE1 (Addgene plasmid number: 32534), pET28a‐LIC UBE2V1 (Addgene Plasmid number: 25619), pOPINF‐OTULIN (Addgene plasmid number: 61464). The pET9‐His‐M1‐linked octa‐ubiquitin, to which the SGCG residues were added in frame after the C‐terminal ubiquitin, was codon‐optimised and gene‐synthesised (Genscript). The pET47b HOIP‐RBR‐LDD (C‐terminal HOIP) and pET28a LIC UBE2L3 were gifts from Dr. Benjamin Stieglitz.^[^ ^54^ ^]^ The pGEX‐CDC34 was a gift from Prof. Dr. Kay Hofmann. ^[^ ^95^ ^]^

**Table 1:** List of primers used for protein mutagenesis.

Mutation	Primers
D474N	fwd: TTACTGTTCTAACTTTCATGCTGAAAG rev: ACTTCCATCTGAGCCCTG
E478G	fwd: TTTTCATGCTGGCAGAGCAGCGAG rev: TCAGAACAGTAAACTTCCATC
Q398X (aa 398–576)	fwd: TAACTCGAGCGGCCGCAT rev: AAGAAGCTTGTTGTGTGTCATCTGTAG
ΔCC 1 (aa 26–119)	fwd: AGGTCATCTGAGGACCCC rev: GGGGGGTCCATTTCCTGT
ΔLIR (aa 169–184)	fwd: GAAGGAGAAGCAGAAGGG rev: CAGCTTGAGCTGCAGTTC
ΔCC 2 (aa 210–454)	fwd: GAAGAGGACCTGGAAACCATG rev: CGTGCCAGTGGAGACTGT
ΔUBAN (aa 454–514)	fwd: ATGGAGATGCAGAGTCGTC rev: CTTGGCAATGGTTTGCTTC
ΔZF (aa 547–576)	fwd: TAACTCGAGCGGCCGCAT rev: TTGCCGCCAGTCCCTGTC
S177E	fwd: CTCAGAAGATGAATTTGTTGAAATTAGGATGGCTGAAGG rev: GAGCCGCTGGAGTTCAGC
S473E	fwd: AGTTTACTGTGAAGATTTTCATGCTGAAAGAGC rev: TCCATCTGAGCCCTGAGG
S513E	fwd: AGGCAGGCAGGAATTGATGGAGATG rev: CCGTCTTCGAAAGCATCATTC
S177A/S473A/513A	S177A fwd:CTCAGAAGATGCCTTTGTTGAAATTAGGATG S177A rev:GAGCCGCTGGAGTTCAGC S473A fwd: AGTTTACTGTGCCGATTTTCATGCTGAAAGAG S473A rev: TCCATCTGAGCCCTGAGG S513A fwd: AGGCAGGCAGGCGTTGATGGAGA S513A rev: CCGTCTTCGAAAGCATCATTC
di‐M1 linked Ubiquitin	fwd:TAACTCGACACTAGTAATAATTTTGTTTAACTTTAAGAAGGAGATAG rev: GCCACCACGCAAGCGCAG
GST‐CFP‐LC3‐His	fwd: CACCACCACTAAGCGGCCGCATCGTGA rev: ATGATGATGCACTGACAATTTCATCCCGAACG

### Cell Lines and Strains

Chemically competent cells NEB 5‐alpha Competent *E. coli* (High Efficiency) (C2987HVIAL, New England Biolabs) were used for cloning steps. For recombinant protein expression, chemically competent *E. coli* BL21(DE3) pLysS cells (L119B, Promega AG, Dübendorf, Switzerland). Mammalian cell lines SH‐SY5Y wildtype (ACC 209, DSMZ‐German Collection of Microorganisms and Cell Cultures, CVCL_0019) and OPTN KO cells (generated in this study from the mentioned SH‐SY5Y wildtype cells) were employed.

### Reagents

Recombinant mono‐ubiquitin (R&D System), undeca‐ubiquitin (11xM1‐ub, Enzo Life Science), 35 mm Glass Bottom and µ‐Slide I Luer (Ibidi, Gräfelfing, Germany), poly‐lysine (Sigma), laminin (Sigma). HOIPIN‐8 (Hycultec GmbH) and TAK243 (Hycultec GmbH), Bafilomycin A (MedChemExpress), GSK8612 (MedChemExpress), Phos‐tag acrylamide (FUJIFILM, Wako), Cy5‐maleimide (Lumiprobe), AF 488‐maleimide (Lumiprobe), 1, 6 hexanediol (Merck). The following primary antibodies were used (**Table** **2**):

**Table 2:** List of the antibodies employed in this study.

Antibody	Source	Identifier
Rabbit monoclonal anti‐linear ubiquitin	Millipore	Cat# MABS199, RRID: AB_2576212
		
Rabbit monoclonal anti‐ Optineurin	Merck	Cat# HPA003279, RRID:AB_1079527
Mouse monoclonal anti ubiquitin	Santa Cruz	Cat# sc‐8017, RRID:AB_628423
Mouse monoclonal anti‐HA	BioLegend	Cat# 901502, RRID: AB_2565007
Rabbit Phospho‐Optineurin (Ser177)	Cell Signaling Technology	Cat# 57548, RRID:AB_2799529
Rabbit monoclonal anti TBK1	Cell Signaling Technology	Cat# 3504, RRID:AB_2255663
Mouse monoclonal anti TBK1	Cell Signaling Technology	Cat# 51872, RRID:AB_2799403
Rabbit monoclonal anti pTBK1	Cell Signaling Technology	Cat# 5483,RRID:AB_2798526
Rabbit monoclonal anti LC3	Cell Signaling Technology	Cat# 3868,RRID:AB_2797680

### Recombinant Protein Expression and Purification

GST‐OPTN constructs were expressed as previously described.^[^ ^34^ ^]^ Briefly, the proteins were expressed in *E. coli* Rosetta pLysS cells (Themofisher). Cells were grown in LB medium at 37 °C until an OD_600_ of 0.4 and then at 18 °C until an OD_600_ of 0.8. Protein expression was induced with 1 mM isopropyl‐β‐d‐thiogalactopyranoside (IPTG) for 16 h at 18 °C. Cells were pelleted and resuspended in a buffer containing 20 mM Tris‐HCl, pH 7.5, 300 mM NaCl, 1 mM DTT, protease inhibitor RNase and DNase. Cells were lysed by French press, and the supernatant was cleared by centrifugation at 30,000 *g* for 30 min at 4 °C and loaded on a GST Prep Fast Flow 16/1(Cytiva) column in an Äkta Pure system (Cytiva). Beads were then washed with low salt (20 mM HEPES, pH 7.5, 300 mM NaCl, and 1 mM DTT) buffer, followed by high salt (20 mM Tris‐HCl, pH 7.5, 500 mM NaCl, and 1 mM DTT) and low salt buffers. GST‐OPTN was eluted (20 mM Tris‐HCl, pH 7.5, 200 mM NaCl, 20 mM L‐glutathione and 1 mM DTT) and dialyzed overnight against 20 mM Tris‐HCl, pH 7.5, 200 mM NaCl, 0.5 mM TCEP. During dialysis, the GST tag was cleaved with TEV protease using a ratio of 1:50 protease:protein. After dialysis, the GST and the His‐TEV were removed with a Glutathione Sepharose 4 Fast Flow (Cytiva) and HisTrap High Performance (Cytiva). The final samples were analysed by SDS‐PAGE and stored at ‐80 °C. The protein concentration was determined using the absorbance at 280 nm and the extinction coefficient of each construct by Nanodrop (Thermo Fisher).

The expression of GST‐TBK1 in insect cells was performed as previously described.^[^ ^94^ ^]^ GST‐CFP‐LC3‐His was expressed in *E. coli* Rosetta pLysS cells. Cells were grown in LB medium at 37 °C until an OD_600_ of 0.8. The protein expression was induced by adding 1 mM IPTG (final concentration) and harvested after 4 h. Cells were pelleted and resuspended in a buffer containing 20 mM Tris‐HCl, pH 7.5, 300 mM NaCl, 1 mM DTT, protease inhibitor and DNase. Cells were lysed by French press, and lysates were cleared by centrifugation at 30,000 g for 30 min at 4 °C. The protein was purified in an Äkta Pure system (Cytiva) and the supernatant was passed in a GSTPrep Fast Flow 16/1 (Cytiva). The column was washed with 10 column volume with equilibration buffer. The elution was performed by 20 mM Tris‐HCl, pH 7.5, 300 mM NaCl, 1 mM DTT, 20 mM L‐glutathione. The protein was subjected to overnight dialysis in 1 L dialysis buffer using a SERVAPOR dialysis tubing membrane 10000 MWCO (Serva, Heidelberg) to reduce the glutathione concentration in 20 mM Tris‐HCl, pH 7.5, 200 mM NaCl, 0.5 mM TCEP. GST‐3C protease was used in a ratio 1:50 protease:protein to cleave the GST. The protein solution is applied to the glutathione‐Sepharose column the next day to remove the cleaved GST. CFP‐LC3‐His samples were checked by SDS‐PAGE after each purification step. The protein concentration was determined using the absorbance at 280 nm and the extinction coefficient of each construct by Nanodrop (Thermo Fisher).

Bacterial expression and purification of GST‐C‐terminal HOIP, GST‐M1‐linked tetra‐ubiquitin, His‐tagged mouse UBE1, His‐M1‐linked octa‐ubiquitin, GST‐CDC34, His‐tagged UBE2V1, GST‐UBE2N and His‐tagged UBE2L3 were performed by standardized methods.^[^ ^54^, ^93^, ^95^ ^]^ The proteins were expressed in *E. coli* BL21 (DE3) pLysS to an OD_600_ of 0.6–0.8 before induction with 0.1 mM IPTG and a change in temperature to 18 °C overnight. In the case of C‐terminal HOIP, the medium was supplemented with 150 µM ZnCl_2_. Cells were harvested and resuspended in 20 mM Tris‐HCl, pH 7.5, 150 mM NaCl, 10% glycerol, and 1 mM DTT and lysed by a French press. After centrifugation for 40 min at 30 000 g, tagged proteins were affinity‐purified with a HisTrap HP nickel column (GE Healthcare) or glutathione Sepharose 4B resin (GE Healthcare). The elution was dialyzed and treated with TEV or precision protease. Another purification step to remove the tag or the protease was implemented as required for each protein by anion exchange (Hi‐Trap Capto Q, Cytiva) or affinity chromatography (HisTrap FF or GST‐trap) or/ and exclusion chromatography (HiLoad 16/600 Superdex 75 pg, Cytiva). The protein concentration was determined using absorbance at 280 nm and the extinction coefficient of each construct by Nanodrop (Thermo Fisher).

### Optineurin Phosphorylation and Fluorescence Labeling

Optineurin was phosphorylated using GST‐TBK1 at 1:250 molar ratio (TBK1:OPTN). The reaction was initiated by adding 1 mM ATP. The sample was incubated at 37 °C for 1.5 h and subsequently overnight at 10 °C. GST‐TBK1 was removed using Glutathione Sepharose 4 Fast Flow (Cytiva Germany GmbH, Dreieich). ATP was removed from the sample using a Zeba desalting column and multiple rebuffering steps with Viaspin columns (Sartorius Stedim Biotech).

To label OPTN with Cy5 fluorophore, the protein was incubated with Cy5‐maleimide in a 2:1 (Cy5:OPTN) ratio overnight at 10 °C with slight agitation. The free Cy5‐maleimide was removed using ZebaTM Spin Desalting Columns 7K MWCO (Thermo Scientific) according to the manufacturer's instructions.

The M1‐linked octa‐ubiquitin contains a cysteine residue in its C‐terminal region. For labelling it was incubated with AF 488‐maleimide in a 2:1(AF 488: 8xM1‐ub) ratio overnight at 10 °C with slight agitation. The free AF 488‐maleimide was removed using ZebaTM Spin Desalting Columns 7K MWCO (Thermo Scientific) according to the manufacturer's instructions.

### Assembly of M1, K63, and K48‐Polyubiquitin and Purification of Hexa‐ and Octa‐Ubiquitin Chains

Free M1‐linked ubiquitin chains were generated by an enzymatic reaction. Briefly, 1 µM E1, 2 µM ubiquitin‐conjugating enzyme E2 UBE2L3, and 4 µM catalytically active C‐terminal HOIP were mixed. Free K63‐inked ubiquitin chains were generated by 1 µM E1, 8 µM UBE2V1, and 8 µM UBE2N6. K48‐inked polyubiquitin were assembled by 1 µM E1 and 25 µM CDC34. The buffer used for the assembly reactions was 40 mM Tris‐HCl, pH 8.5, 0.5 mM DTT. The reaction mixture contained 100 µM ubiquitin (R&B Systems) and was started by adding ATP/Mg^+2^ to a final concentration of 10 mM. The mixture was incubated for 2 h (or as indicated in the figures) at 37 °C for M1‐linked ubiquitin, 3 h at 30 °C for K63‐linked ubiquitin and at room temperature overnight for K48‐linked ubiquitin. The chains generated were treated with sodium acetate pH 4.5 to reach a final concentration of 50 mM. This low pH ensured that the enzymes were unfolded and not active, while the ubiquitin chains were stable in solution. Then, the samples were centrifuged and re‐buffered in 20 mM Tris‐HCl, pH 7.4, 0.5 mM TCEP to remove ATP using Viaspin columns (Sartorius Stedim Biotech), reaching the initial volume of reaction. As a control, the reaction was performed without ATP.

The assembly of K48‐linked and K63‐linked octa‐ubiquitin chains was performed as described above, but the preparation was scaled to a final volume of 1 mL with 10 mg of ubiquitin. After the reaction and acidification, the samples were diluted in 50 mM acetate buffer in a final volume of 10 mL and centrifuged at 20 000 g. The supernatant was collected, and the different ubiquitin chains were purified using a Resource S column (Cytiva) in acetate buffer 50 mM sodium acetate, pH 4.5. A gradient from 0 to 500 mM NaCl was performed on 350 mL and each peak was collected and analysed by SDS‐PAGE followed by Coomassie staining. The ubiquitin chains were collected and adjusted to a neutral pH. Then, the proteins were rebuffered and stored at −80 °C.

### SDS‐PAGE and Immunoblotting

The proteins were separated busing SDS‐PAGE (4 – 20% gradient gel, BioRad) or Phos‐tag gels. Transference was performed by electroblotting onto nitrocellulose membrane (Mini Trans‐Blot Electrophoretic Transfer Cell, Bio‐Rad, Hercules, CA, USA). The nitrocellulose membranes were blocked with 5% non‐fat dry milk in TBST (TBS with 0.1% Tween 20) for 60 min at room temperature and then incubated with the primary antibody diluted in blocking buffer overnight at 4 °C. Following a series of washes with TBST, the membranes were incubated with horseradish peroxidase‐conjugated secondary antibody for 60 min at room temperature. Following further washing with TBST, the antigen was detected by the enhanced chemiluminescence (ECL) detection system (Promega) using the Azure Sapphire Biomolecular Imager (Azure Biosystems, USA) according to the manufacturer's instructions.

### Phase Separation Assays

Protein aliquots were thawed on ice and using Vivaspin 500 columns of 30 or 10 kDa molecular weight (Sartorius Stedim Biotech, Göttingen), the buffer was exchanged by centrifugation six times for 10 min at 10,000 g at 4 °C to 20 mM Tris‐HCl, pH 7.4, 150 mM NaCl, 0.5 mM TECP. Any precipitates were removed by centrifugation at 20,000 g for 15 min at 4 °C. Unless otherwise stated, Optineurin and p‐Optineurin were analyzed at a concentration of 5 µM. For Optineurin phase separation in the presence of polyubiquitin generated by in vitro reactions, a final ubiquitin concentration of 40 µM was used in each experiment. In the case of mono‐ubiquitin, tetra‐, octa‐, and undeca‐ubiquitin, a final concentration of 25 µM final was used. Concentrations that were different from the above are indicated in the figures. To analyze the phase separation of Optineurin, 12 µL of each sample was placed onto a 35 mm glass bottom dish (Ibidi, Gräfelfing) and incubated for 1 h before imaging.

### Sedimentation Assay

For the sedimentation assay, pOPTN (5 µM) and ubiquitin chains (40 µM) were incubated for 1 h at room temperature. Then, the samples were centrifuged at 21 000 g for 30 min in a tube. After centrifugation, the supernatant and pellet were separated into two tubes. The pellet fraction was washed once with a buffer solution containing 20 mM Tris‐HCl, pH 7.4, 150 mM NaCl, and 0.5 mM TCEP. Supernatant and pellet fractions (1:5 ratio) were analyzed SDS/PAGE followed by immunoblotting.

### Giant and Small Unilamellar Vesicle Preparation

50 µL of 5% PVA (Poly[1‐(acetyloxy)ethylene]) was added to a coverslip (Ibidi, Gräfelfing) and dried at 90 °C. Then, 98% POPC (1‐palmitoyl‐2‐oleoyl‐sn‐glycero‐3‐phosphocholine), 2% DGS NTA‐Ni (1,2‐dioleoyl‐snglycero‐3‐[(N‐(5‐amino‐1‐carboxypentyl)iminodiacetic acid)succinyl], nickel salt), and 0.15% rhodamine DHPE (Invitrogen Lissamine Rhodamine B 1,2‐dihexadecanoyl‐sn‐glycero‐3 phosphoethanolamine, triethylammonium salt) were dissolved in 100 µL chloroform and added to the dried PVA. This mixture was dried again at high temperature, then 500 µL 10 mM HEPES pH 7.5 buffer was added, and the solution was incubated at 37 °C for 20 min to enable proper lipid hydration and GUVs formation reaching a final lipid concentration of 3 mg mL^−1^. Freshly prepared GUVs were used in each experiment. The GUVs were then incubated with LC3 (2.5 µM) and deposited on poly‐lysine coated channel slides (µ‐Slide ILuer, Ibidi). The GUVs were washed with 20 mM Tris‐HCl, pH 7.4, 150 mM, 0.5 mM TCEP and incubated with purified proteins as indicated. After 60 min, fluorescence microscopy was performed.

POPC (1‐palmitoyl‐2‐oleoyl‐sn‐glycero‐3‐phosphocholine), DGS NTA‐Ni (1,2‐dioleoyl‐sn‐glycero‐3‐[(N‐(5‐am ino‐1‐carboxypentyl)iminodiacetic acid)succinyl], nickel salt) stock solutions were prepared in chloroform and stored at 4 °C. To prepare SUVs, the components were mixed and lyophilized overnight. The components were dissolved in 20 mM Tris‐HCl, pH 7.4, 150 mM NaCl, and 0.5 mM TCEP to a final lipid concentration of 15 mg mL^−1^. The mixture was passed through a membrane (PC Membranes 0.05 µm, Avanti Polar Lipids) at least 21 times, using an extruder (Extruder Set with Holder/Heating Block, Avanti Polar Lipids) at 35 °C. The SUVs were prepared at a final concentration of 3 mg mL^−1^ and incubated for 1 h with CFP‐LC3 ‐His at a concentration of 5 µM. Then, 11xM1‐ub (15 µM) and p‐OPTN (5 µM) were added and incubated for 1 h.

### Fluorescence Microscopy and Image Processing

Fluorescence microscopy was performed using a Zeiss ELYRA PS.1 system equipped with an LSM880 (Carl Zeiss, Oberkochen) and a 20x/0.8, 63x/1.4 oil or 100x/1.46 oil immersion objective or a C2+ system (Nikon). Super‐resolution images of cells were generated by the Structured Illumination (SIM) technology using 405, 488, 561, and 647 nm widefield laser illumination. SIM confocal images were processed using the ZEN Black software (Carl Zeiss, Oberkochen), and image data were exported using the ZEN Blue software for further analysis. Laser scanning microscopy was performed using the lasers 405, 488, 561 nm, and 647 nm set in individual channels to avoid cross‐talk. The pinhole was adjusted to generate an optical section of 2–5 µm; the acquisition settings were kept constant throughout the experiment. The condensate visualization was performed using a 63× oil immersion objective. A 63× NA 1.4 oil immersion objective was used to record a z‐stack of 44.9 × 44.9 × 2.25 µm volume. The argon laser power was set to 15% at 405 nm, 3% at 488 nm, 0.006% at 561 nm and 5% at 647 nm with pixel dwell time of 5.71 µs. Laser power, gain and field of view were kept constant during all measurements.

ImageJ and IMARIS 10.1.2 were used for further image processing. OriginPro and GraphPad were used to analyze and data plotting.

### 3D‐Image Reconstruction and Co‐Localization Analysis

Images stacks in z were collected on a Zeiss ELYRA PS.1 microscope using the Leap‐function and the minimal interval. Images were processes using the Zeiss Zen software and imported into Imaris 10.0.3 image analysis software. 3D surfaces of condensates and GUVs were reconstructed using the Surface module in batch mode. Surface‐to‐surface contact sites were analysed using a MatLab Plugin (Imaris website modules). Co‐localization was analysed using the Zeiss Zen Black software to obtain the Pearson correlation coefficient.

### Fluorescence Recovery after Photobleaching (FRAP)

For FRAP, 5 consecutive bleaching pulses using the 405, 488, 564, and 614 nm lasers were performed within a defined region of interest. Three circular areas were defined as regions of interest (ROI). One ROI was bleached with 100% laser power on a condensate, while the other two ROIs were chosen as reference and background signals, respectively. The measured fluorescence signal of the bleached ROI was corrected with the measured signal of the reference and background ROI. Fluorescence recovery was measured every 20 ms for 1–3 min and plotted as a percentage of baseline fluorescence.

### Isothermal Titration Calorimetry

Isothermal titration calorimetry (ITC) experiments were conducted using MicroCal PEAQ‐ITC (Malvern). The protein solutions were first dialyzed in (Slide‐A‐Lyzer MINI Dialysis Device, 3.5K MWCO, 0.5 mL, Thermo Scientific) against 20 mM Tris‐HCl, pH 7.4, 150 mM NaCl, and 0.5 mM TCEP at 4 °C for 12 h with gentle agitation. The calorimeter cell and injection syringe were extensively rinsed with buffer. Wildtype OPTN and its variants were placed in the cell, and di‐M1‐ubiquitin was added by the syringe. 19 incremental titrations (1 × 0.4 µL followed by 18 × 2 µL) with 150 s intervals at 25 °C and a stirring rate of 750 rpm were performed. The data was processed using the MicroCal PEAQ‐ITC Analysis Software v1.41 (Malver), with fixed integration intervals and manually checked baselines.

### Negative Staining Electron Microscopy

Optineurin, phosphorylated Optineurin and phosphorylated Optineurin in phase separation droplets with 11xM1‐ub were prepared at the concentrations indicated. Carbon formvar‐coated hexagonal copper grids were prepared as sample carrier by glow discharge. 3.0 µL of the sample were applied to the grids and incubated for 1 min before blotting by side‐blot method with Whatman paper. Subsequently, the grid was washed 2x with 3 µL of sample buffer and 1x with 3 µL of 2% uranyl acetate. Finally, the sample was incubated with 3 µL of 2% uranyl acetate for 1 min, blotted and the grid allowed to dry. Examination of negative stain samples was performed on a Talos L120C G2 transmission electron microscope (Thermo Fisher Scientific), operated at 120 kV and equipped with a 4k x 4k Ceta 16 M CEMOS camera using TEM Imaging & Analysis Software (Thermo Fisher Scientific).

### Cryo‐Electron Tomography of Optineurin Reconstituted with LC3‐Decorated Liposomes

Vitrification of phosphorylated Optineurin was reconstituted with LC3‐decorated liposomes and 11xM1‐ub, and all controls were performed with a Leica GM2 plunge freezer (Leica). The environmental settings in the chamber were set to 10 °C and 80% humidity with a 60% GN_2_‐flow. 3.6 µL of sample were applied onto glow‐discharged Quantifoil Cu 200 mesh R2/R1 grids. Back‐side blotting was performed for 3.0 s with the sensor blotting option and 2.0 mm additional move before plunging into liquid ethane.

Tomograms were collected at a Titan Krios 300 keV transmission electron microscope equipped with a BioContinuum energy filter and K3 camera at 64,000x using Tomo5 software (Thermo Fisher Scientific). The acquisition followed a dose‐symmetric bidirectional acquisition scheme ranging from ‐60° to 60° with a 3° increment. Each tilt was collected in six frames with an average dose of 3.0 e^−^/Å^2^/tilt. The total dose was approximately 120 e‐/Å^2^ for the whole tilt series. Between the tomograms the defocus was varied between ‐2.0 to ‐4.0 µm. The raw data was motion corrected with MotionCorr2 and tomograms reconstructed with AreTomo.^[^ ^96^, ^97^ ^]^ Lastly, the tomograms were denoised using cryoCARE.^[^ ^98^ ^]^ The resulting pixel size after processing was 10.88 A/pix after 16x binning in the end.

### Generation of OPTN CRISPR/Cas9 Knockout (KO) SH‐SY5Y Cells

sgRNAs (OPTN:ConCUAAAUAAUCAAGCCAUGAA; GAUUUGAGGAGCUUUCGGCC) were designed using the Synthego website (www.design.synthego.com). 1.5 nmol sgRNAs were rehydrated in 50 µL nuclease‐free 1× TE buffer (10 mM Tris‐HCl, 1 mM EDTA, pH 8.0) to a final concentration of 30 µM (30 pmol µL^−1^). sgRNA and recombinant CAS9 were delivered as ribonucleoprotein (RNP) complexes using a 4D‐Nucleofector X‐Unit (Lonza). Briefly, for the assembly of the RNP complexes, Cas9 2NLS and sgRNAs were combined in Nucleofector solution at a molar ratio of 9:1 sgRNA to Cas9 and incubated for 10 min at room temperature. The cells were resuspended at a concentration of 150000 cells/5 µL. 5 µL of the cell suspension was added to the 25 µL of pre‐complexed RNPs for a total transfection volume of 30 µl per reaction and transferred to Nucleofector cartridges. Nucleofection was performed according to the predefined protocol (CA‐137 for SH‐SY5Y) and cells were carefully resuspended in each well of the Nucleocuvette with 70 µl of pre‐warmed growth medium and transferred to the pre‐warmed 6‐well and incubated in a humidified 37 °C/5% CO_2_ incubator. After 24 h the medium was replaced.

For clone screening, the cells were split into two 6‐well cell culture plates, and pools were analyzed by PCR and subsequent DNA sequencing. For this, primer pairs (fwd: GCGGTACCCAAATCCACTTT; rev: ACAATGGATCGGTCTGCTCA) were used extending ≈ 200–250 bp 3´and 5´ of the sgRNA binding region. To perform cell pool or single clone sequencing analysis, genomic DNA was isolated using a genomic DNA extraction kit (Monarch Genomic DNA Purification Kit, New England Biolabs, Frankfurt am Main, Germany) and the PCR was optimized to yield a single amplicon. Following PCR product purification (NucleoSpin Gel and PCR Clean‐up, Macherey‐Nagel GmbH, Düren, Germany), the DNA was sent for Sanger sequence analysis (Microsynth Seqlab GmbH, Göttingen, Germany). The KO efficiency of the cell pools and single colony clones was determined using the SYNTHEGO ICE analysis website (https://ice.synthego.com). To isolate single KO clones, the KO cell pools were diluted to 1 cell/100 µl and 5 cells/100 µl, and the dilutions were distributed over several 96‐well plates. 15–25 clones were grown from single cells and reanalyzed using the above‐mentioned process. Finally, clones with a high KO score were amplified and KO efficiency was confirmed by immunoblotting.

### Mammalian Cell Culture and Transfection

SH‐SY5Y wildtype (ACC 209, German collection of microorganisms) and OPTN KO cells (generated in this study from SH‐SY5Y wildtype) were cultured in Dulbecco's modified Eagle's medium F‐12 (DMEM/F12) supplemented with 15% (v/v) fetal bovine serum (FBS), 100 IU mL^−1^ penicillin, 100 µg mL^−1^ streptomycin sulfate (1% P/S) and 1% non‐essential amino acids solution. For the analysis, cells were transfected using lipofectamine and Plus reagent (Thermo Fischer) and jetOPTIMUS (Sartorius) as specified by the manufacturer. Cells contamination was not detected during the study.

### Immunocytochemistry

Cells were cultivated on glass coverslips (Laboratory Glassware Marienfeld). For some experiments, coverslips were coated with poly‐L‐lysine (PLL) (Sigma) or with PLL and laminin (Sigma). 24 h after seeding the cells were fixed for 10 min with 4% paraformaldehyde in PBS or Tris‐HCl pH 7.4, and permeabilized and blocked in 0.2% (v/v) Triton X‐100, 5% (v/v) goat serum in PBS for 2 h. Cells were stained with primary antibodies at a dilution of 1:100 to 1:1000 in 0.2% (v/v) Triton X‐100, 5% (v/v) goat serum in PBS or Tris‐HCl at 4 °C overnight, washed 3 times with PBS and incubated with fluorescent dye‐conjugated secondary antibodies Alexa Fluor 488, Alexa Fluor 555 and or Alexa Fluor 647 (Thermo Scientific) as corresponds, at a dilution of 1:1000 for 1 h at room temperature. After extensive washing, the cells were mounted in Fluoroshield G (Thermo Scientific) containing DAPI (Sigma).

### Bioinformatic Analysis

The structural characteristics were evaluated using IUPred,^[^ ^50^ ^]^ Fuzdrop,^[^ ^51^ ^]^ and CIDER.^[^ ^53^ ^]^

### Quantification and Statistical Analysis

Data represent the mean ± SD, n numbers are indicated in the figure legends. For the quantification analysis in which manual counting was used, not all experiments were performed in a blinded manner. All statistical analyses were performed using GraphPad PRISM (Version 8; San Diego, CA, USA) and OriginPro (Version 2023). To check for Gaussian distribution of the data, the Kolmogorov–Smirnov test was applied. Appropriate parametric and non‐parametric tests were chosen based on the outcome of the test. For comparing more than 2 parametric datasets, one‐way ANOVA was applied. To correct for α‐error inflation resulting from multiple comparisons, ANOVA was followed by Tukey's post hoc multiple comparison tests. For the direct comparison of two non‐parametric datasets, the Wilcoxon Mann–Whitney (*U*‐test), and the comparison of more than 2 non‐parametric datasets, the Kruskal–Wallis test was used. Significance levels for all tests: *p  ≤ 0.05; **p  ≤ 0.01; ***p ≤ 0.001. Representative images are displayed for the analysis of microscopic images; experiments were performed at least three times.

## Conflict of Interest

S.M. is a member of the scientific advisory board of Casma Therapeutics. The other authors declare no conflict of interest.

## Author Contributions

Conceptualization: M.G.H., J.T., C.S. and K.F.W. Validation: M.G.H., L.K., L.J., V.B., L.J.K., D.K., J.T., C.S. and K.F.W. Visualization: M.G.H., L.K., L.J., V.B., L.J.K. and D.K. Writing‐original draft: M.G.H., J.T., C.S. and K.F.W. Writing‐review & editing: E.A. and S.M. Formal analysis: M.G.H., L.K., L.J. and V.B. Methodology: M.G.H., L.J. and V.B. Investigation: M.G.H., L.K., L.J., V.B., L.J.K. and D.K. Data curation: M.G.H. and V.B. Managed resources: E.A., S.M., and C.S. Supervision: C.S., J.T. and K.F.W. Funding acquisition and project administration: K.F.W.

## Supporting information



Supporting Information

## Data Availability

The data that support the findings of this study are available from the corresponding author upon reasonable request.
