# Supplementary material for: TBK1 Induces the Formation of Optineurin Filaments That Condensate with Polyubiquitin and LC3 for Cargo Sequestration
Source: Adv Sci (Weinh). 2025 Dec 17;13(16):e09927. doi: 10.1002/advs.202509927 (PMC13042857; doi:10.1002/advs.202509927)
Supplement: Supplementary file 1 — Supporting Information [file ADVS-13-e09927-s001.docx]

Supporting Information

TBK1 Induces the Formation of Optineurin Filaments that Condensate with Polyubiquitin and LC3 for Cargo Sequestration

Maria Georgina Herrera, Lena Kühn, Lisa Jungbluth, Verian Bader, Laura J. Krause, David Kartte, Elias Adriaenssens, Sascha Martens, Jörg Tatzelt, Carsten Sachse, and Konstanze F. Winklhofer*

^*^Corresponding author: Konstanze F. Winklhofer, Email: [Konstanze.Winklhofer@ruhr-uni-bochum.de](mailto:Konstanze.Winklhofer@ruhr-uni-bochum.de)


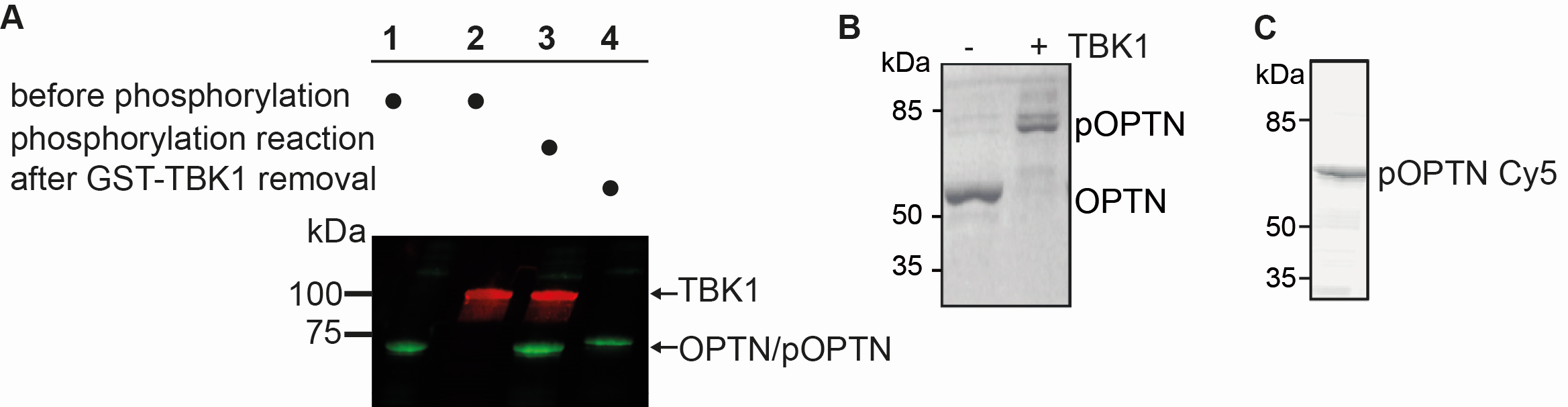


**Figure S1. Phosphorylation and labelling of phosphorylated Optineurin.**

**A**. Immunoblot analysis of the different purification steps of phosphorylated Optineurin**.** Lane 1: recombinantly expressed Optineurin before TBK-1 mediated phosphorylation; lane 2: GST-TBK1; lane 3: mixture of TBK1 and pOPTN after the phosphorylation reaction; lane 4: phosphorylated Optineurin after the removal of TBK1 by GST-Trap affinity chromatography and buffer exchange.

**B.** Phos-tag^TM^ gel showing non-phosphorylated Optineurin (OPTN) and Optineurin phosphorylated by TBK1 (pOPTN).

**C.** SDS- PAGE of pOPTN labeled with Cy5 detected by fluorescence.


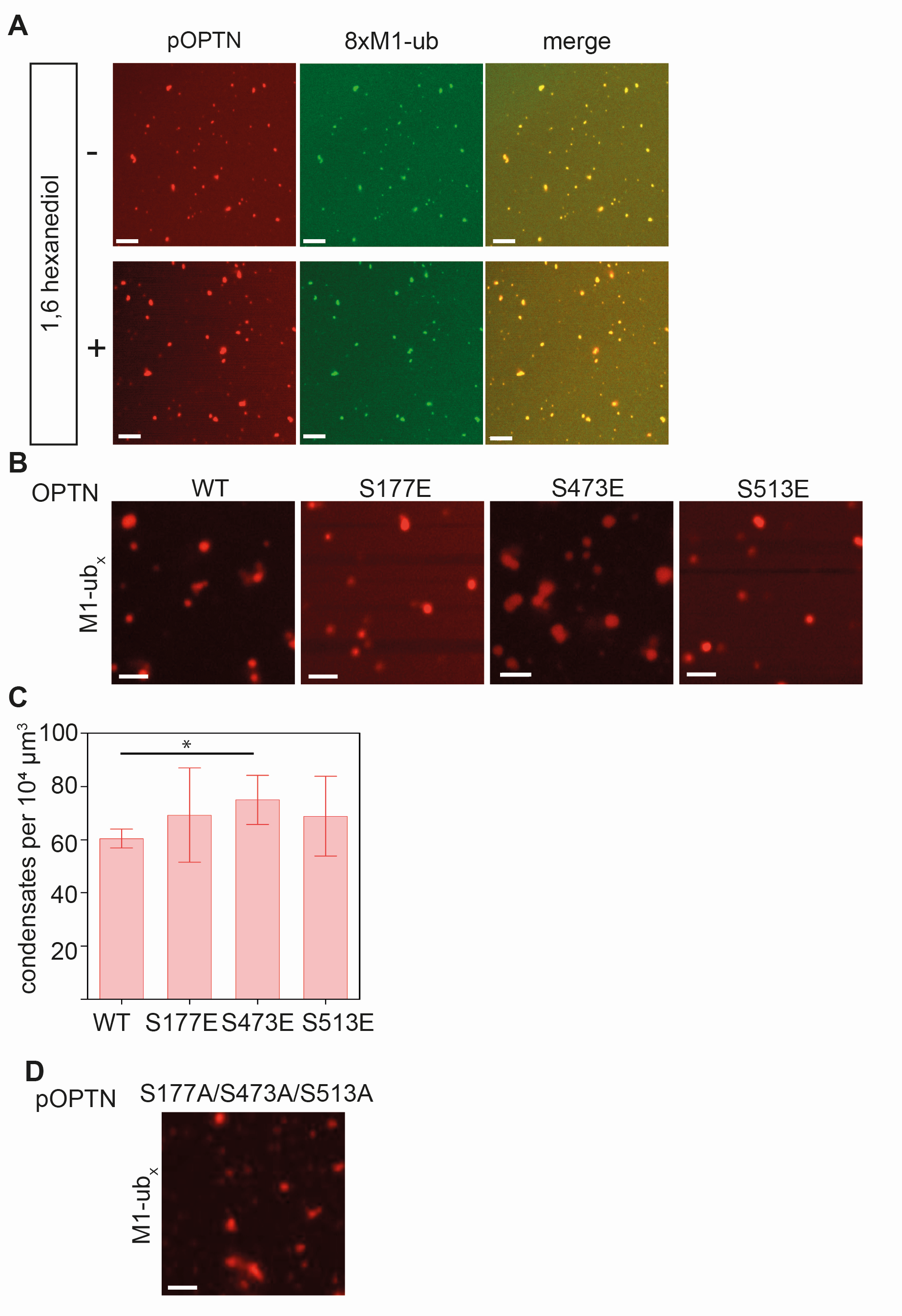


**Figure S2. Phosphorylated Optineurin condensates are insensitive to 1,6-hexanediol, and phase separation of Optineurin requires its phosphorylation on multiple residues.**

**A.** Cy5-labelled pOPTN (5 μM) was mixed with AF 488-labelled 8xM1-ub (50 μM). After 60 min, the sample was treated with 5% 1,6-hexanediol for 30 minutes and then analysed by fluorescence microscopy. Scale bar: 3μm

**B.** Wildtype (WT) Optineurin or the phosphomimetic mutants S177E, S473E or S513E were incubated with M1-ubx for 60 min and then analysed by fluorescence microscopy. Scale Bar: 3 μm.

**C.** Quantification of the number Optineurin condensates formed in the presence of M1-ub_x_ corresponding to the experiment shown in B. Data represent the mean ± SD of five independent experiments. The statistical analysis was performed using a Mann-Whitney test (**p* = 0.0436, n = 5).

**D.** The triple serine Optineurin mutant S177A/S473A/S513A was phosphorylated by TBK1. After removal of TBK1, the mutant was incubated with M1-ub_x_ and analysed by fluorescence microscopy. Scale Bar: 3 μm.


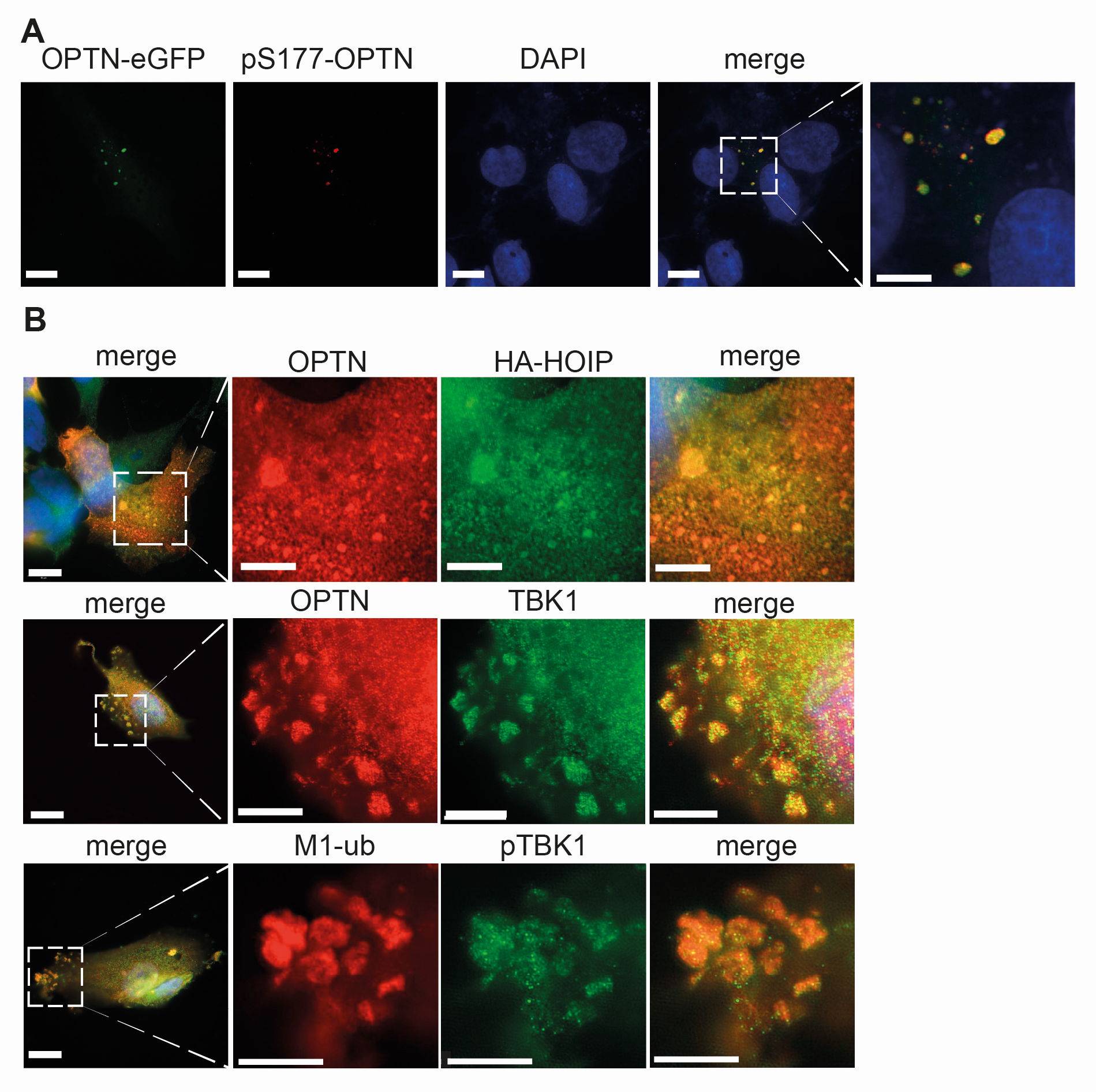


**Figure S3. Optineurin foci formed in SH-SY5Y cells upon HOIP overexpression stain positive for Optineurin, TBK1, p-TBK1 and M1-linked ubiquitin.**

**A.** OPTN-KO SH-SY5Y cells transiently transfected with OPTN-eGFP were analysed by immunocytochemistry and fluorescence microscopy 24 h after transfection using a pS177-specific Optineurin antibody. Scale bar: 10 µm; zoom-in scale bar: 5 µm.

**B.** WT SH-SY5Y cells transiently transfected with HA-HOIP were analysed by immunocytochemistry and fluorescence microscopy 24 h after transfection using antibodies against HA, Optineurin, TBK1, p-TBK1, and M1-ubiquitin. Scale bar: 10 µm, zoom-in scale bar: 5 µm.


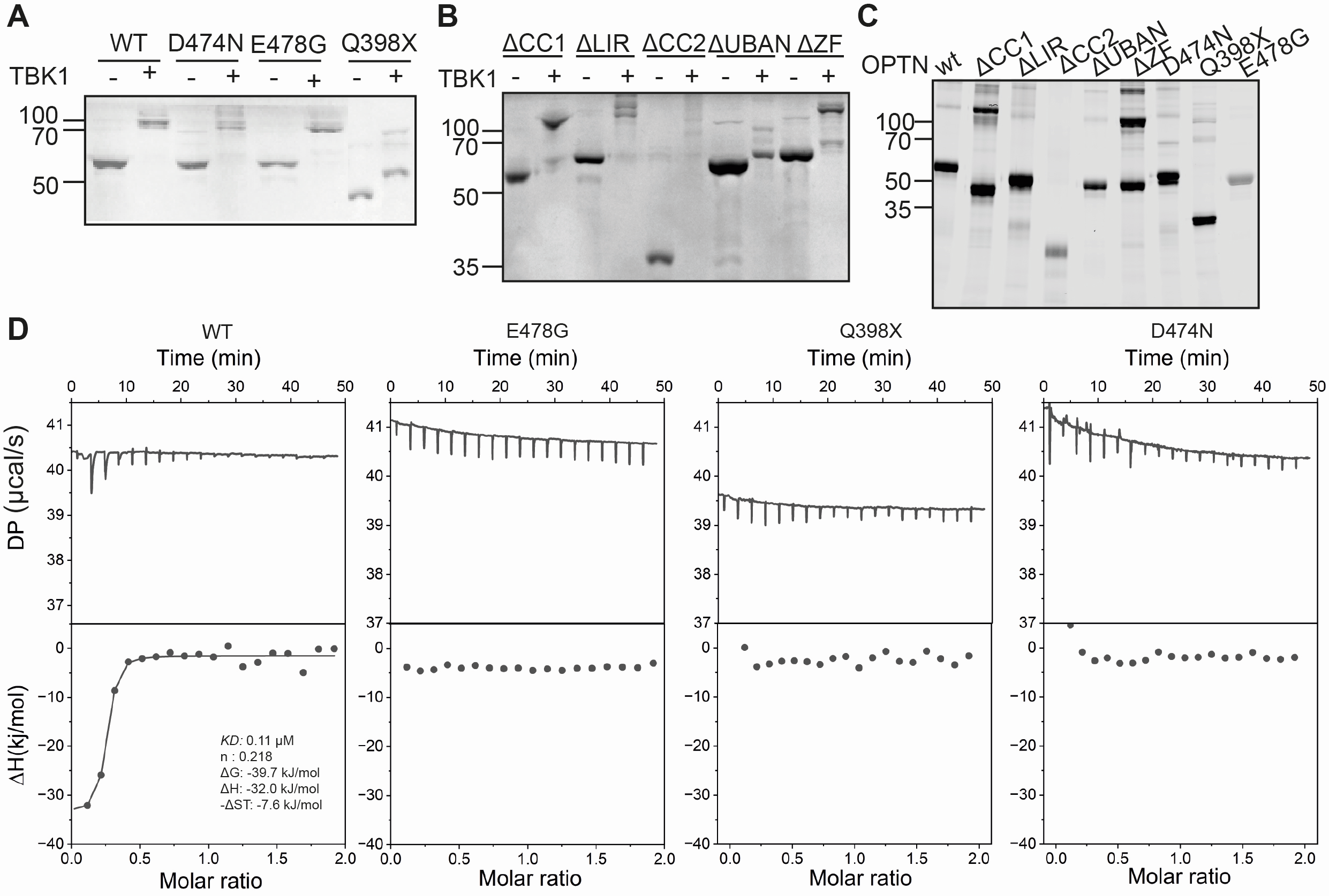


**Figure S4. Analysis of recombinant wildtype Optineurin and Optineurin variants**.

**A, B:** Phosphorylation of wildtype (WT) and Optineurin variants by TBK1. Shown are representative Phos-tag™ gels (Coomassie staining) of Optineurin UBAN variants (A) and domain deletions (B) before and after phosphorylation with TBK1.

**C.** SDS-PAGE analysis of the Optineurin variants phosphorylated and labelled with Cy5-maleimide. The gel was visualized using fluorescence detection.

**D.** Isothermal titration calorimetry (ITC) of Optineurin variants and M1-linked di-ubiquitin. The raw thermograms are plotted in the upper panel. The ITC isotherms, plotted as a function of the titrant-to-analyte molar ratio (Optineurin:M1-linked ubiquitin), are shown in the bottom panel. The data were fitted to a one-site binding model (solid line) on the horizontal axis.


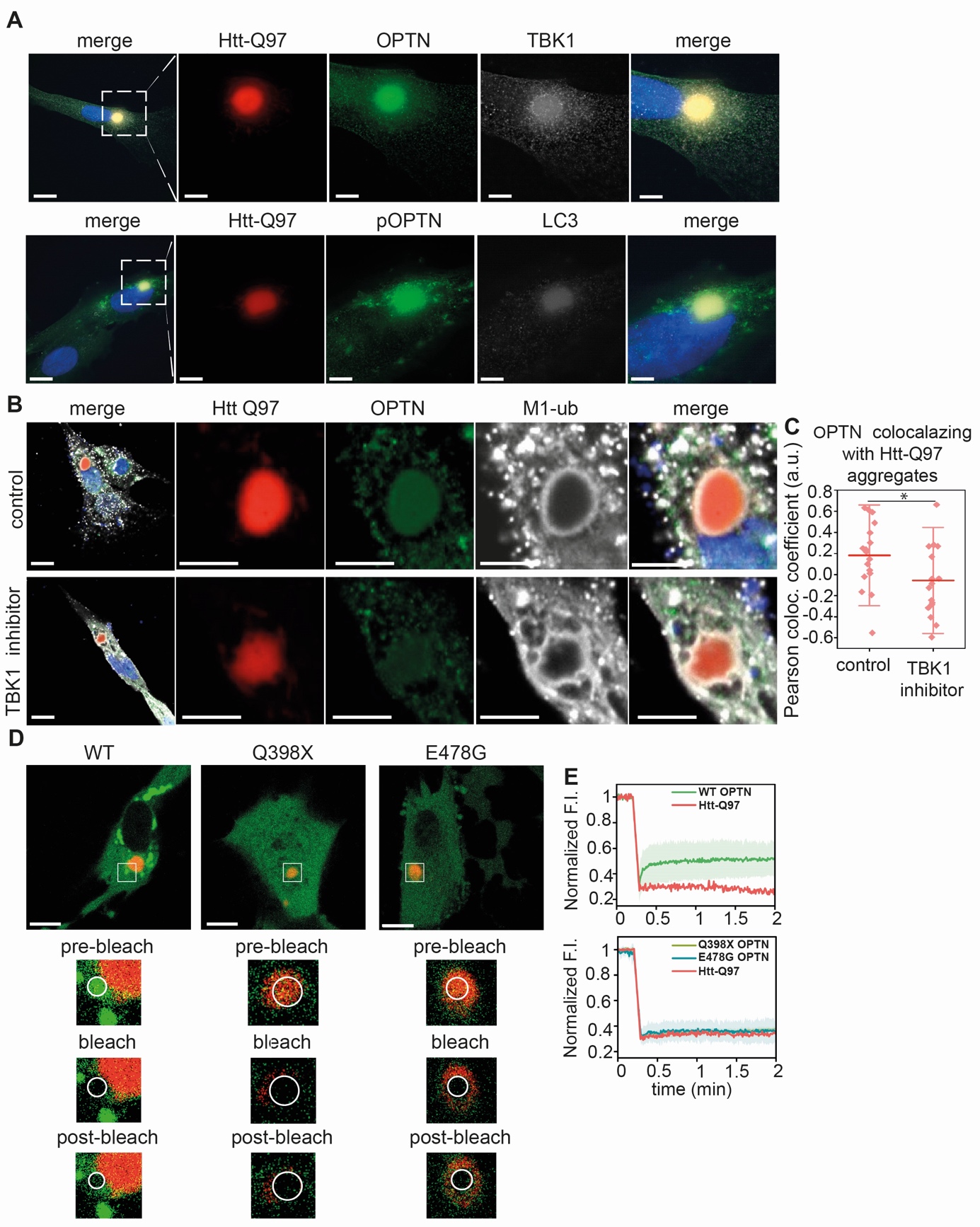


**Figure S5. Effects of TBK1 inhibition and pathological UBAN mutations on the recruitment of Optineurin to Htt-Q97 aggregates**

**A.** SH-SY5Y cells transiently transfected with Htt-Q97-mScarlet were analysed by immunocytochemistry and structured illumination fluorescence microscopy 24 h after transfection using antibodies against Optineurin, p-Optineurin, TBK1, LC3 and M1-ubiquitin. Scale bar: 10 µm; zoom-in scale bar: 5 µm.

**B.** SH-SY5Y cells transiently transfected with Htt-Q97-mScarlet were treated for 24 h with TBK1 inhibitor GSK8612 (10 μM). Control and TBK1 inhibitor-treated cells were in addition treated with bafilomycin A (25 nM) for 24 h to prevent autophagosomal degradation of Htt-Q97-mScarlet and allow visualization of the aggregates. The samples were fixed and analysed by immunocytochemistry and fluorescence microscopy using antibodies against Optineurin and M1-ubiquitin. Scale bar: 10 µm, zoom-in scale bar: 3 µm.

**C.** Quantification of the colocalization of Optineurin with Htt-Q97-mScarlet using the Pearson colocalization coefficient. Data are displayed as mean ± SD, n = 16 individual cells. Statistics: two-tailed Mann–Whitney *U*-test. * *p* = 0.4.

**D.** Live cell imaging and FRAP analysis of OPTN-KO SH-SY5Y cells transiently transfected with WT, Q398X or E478G OPTN-eGFP and Htt-Q97-mScarlet 24 h after transfection. Scale bar: 10 µm.

**E.** Fluorescence recovery after photobleaching (FRAP) of wildtype OPTN-eGFP or mutant Q398X and E478G Optineurin-eGFP at Htt-Q97-mScarlet aggregates was performed by 5 consecutive bleaching pulses using the 488 nm and 561 nm lasers at 100% intensity within a defined region of interest (white circles) at Htt-Q97-mScarlet aggregates (red). Fluorescence recovery was measured for 2 min and plotted as a percentage of baseline fluorescence.


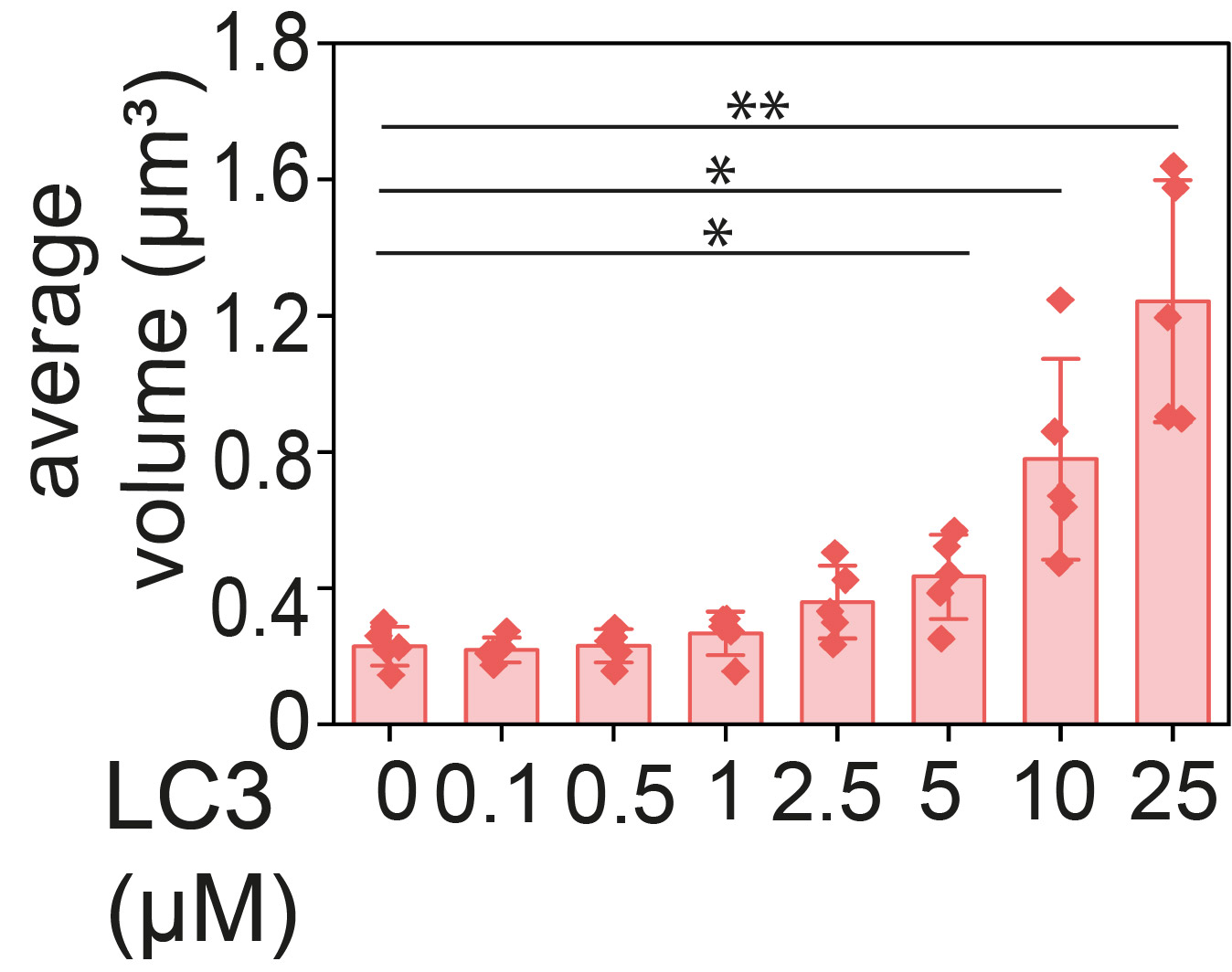


**Figure S6. LC3 increases the average volume of pOPTN/M1-ub_x_ condensates in a concentration-dependent manner.**

Purified LC3 was incubated with pOPTN/M1-ub_x_ (5 μM/40 μM) and fluorescence microscopy was performed after 60 min. Quantification of the average volume of pOPTN condensates corresponding to the conditions shown in Figure 5B. Data represent the mean ± SD of five independent experiments. The statistical analysis was performed using a Mann-Whitney test (***p* ≤ 0.01).


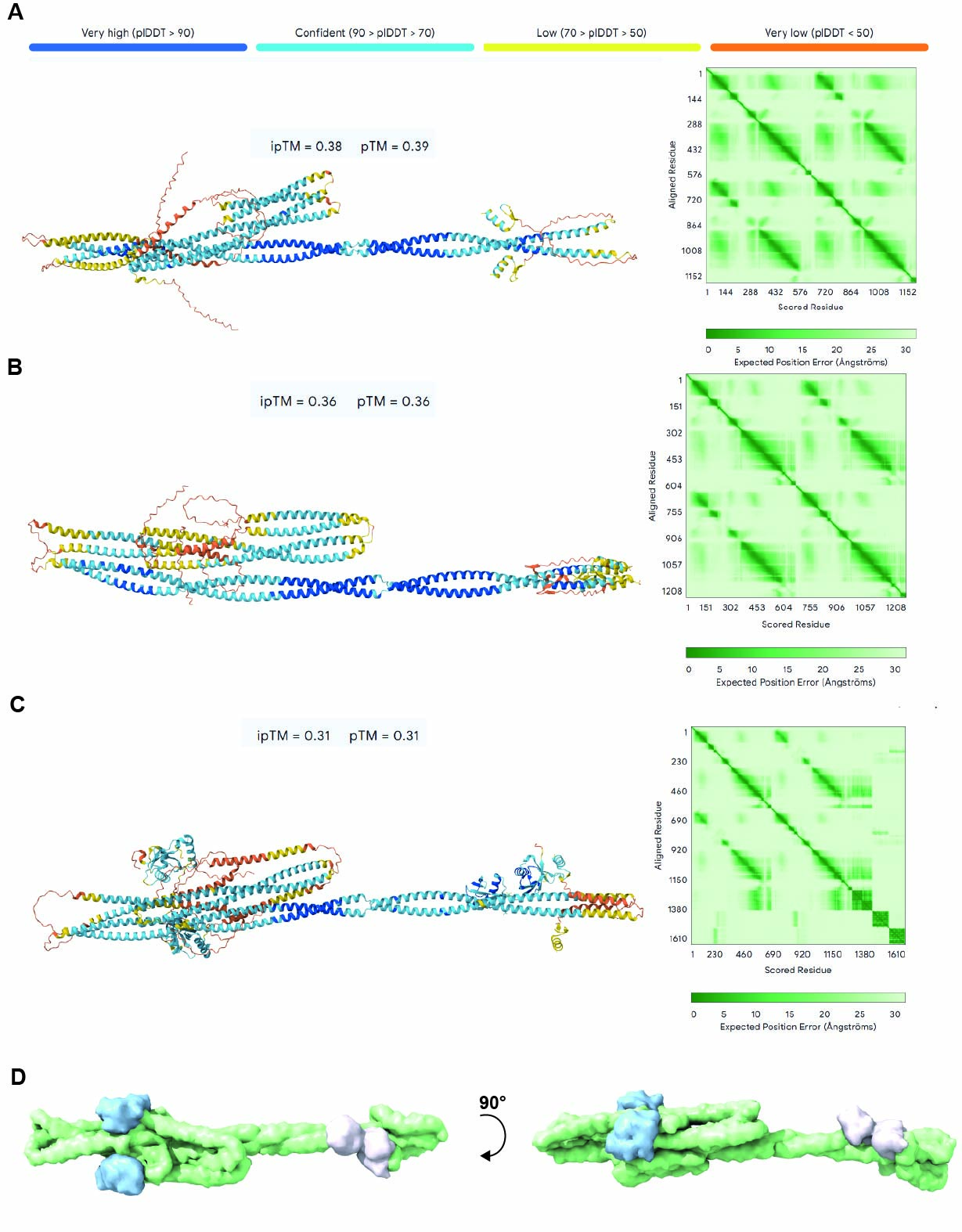


**Figure S7. AlphaFold 3 prediction of Optineurin structural organization.**

**A.** Model of the Optineurin dimer. The model was colored according to a per-atom confidence estimate. Predicted aligned error (PAE) plot reveals spatial proximity of the domains.

**B.** Model of the Optineurin dimer phosphorylated at residues S177, S473, and S513. The model was colored according to a per-atom confidence estimate. Predicted aligned error (PAE) plot reveals the spatial proximity of the domains.

**C.** Model of the Optineurin dimer phosphorylated at residues S177, S473, and S513 interacting with M1-linked di-ubiquitin and two molecules of LC3. The model was colored according to a per-atom confidence estimate. Predicted aligned error (PAE) plot reveals the spatial proximity of the domains.

**D.** Model of the Optineurin dimer phosphorylated at residues S177, S473, and S513 interacting with di-ubiquitin and two molecules of LC3. The AlphaFold 3 predictions rendered at 10 Å resolution with Optineurin in green, LC3b in blue and ubiquitin in grey.
